# DOT1L promotes progenitor proliferation and primes neuronal layer identity in the developing cerebral cortex

**DOI:** 10.1093/nar/gky953

**Published:** 2018-10-17

**Authors:** Henriette Franz, Alejandro Villarreal, Stefanie Heidrich, Pavankumar Videm, Fabian Kilpert, Ivan Mestres, Federico Calegari, Rolf Backofen, Thomas Manke, Tanja Vogel

**Affiliations:** 1 Institute of Anatomy and Cell Biology, Department of Molecular Embryology, Medical Faculty, Albert-Ludwigs-University Freiburg, 79104 Freiburg, Germany; 2 Bioinformatics Group, Department of Computer Science, Albert-Ludwigs-University Freiburg, 79110 Freiburg, Germany; 3 Max Planck Institute of Immunobiology and Epigenetics, 79108 Freiburg, Germany; 4 DFG-Research Center and Cluster of Excellence for Regenerative Therapies (CRTD), School of Medicine, Technical University Dresden, 01307 Dresden, Germany; 5 Centre for Biological Signalling Studies (BIOSS), Albert-Ludwigs-University Freiburg, 79104 Freiburg, Germany; 6 Centre for Biological Systems Analysis (ZBSA), Albert-Ludwigs-University Freiburg, 79104 Freiburg, Germany; 7 Center for non-coding RNA in Technology and Health, University of Copenhagen, DK-1870 Frederiksberg C, Denmark

## Abstract

Cortical development is controlled by transcriptional programs, which are orchestrated by transcription factors. Yet, stable inheritance of spatio-temporal activity of factors influencing cell fate and localization in different layers is only partly understood. Here we find that deletion of *Dot1l* in the murine telencephalon leads to cortical layering defects, indicating DOT1L activity and chromatin methylation at H3K79 impact on the cell cycle, and influence transcriptional programs conferring upper layer identity in early progenitors. Specifically, DOT1L prevents premature differentiation by increasing expression of genes that regulate asymmetric cell division (*Vangl2, Cenpj*). Loss of DOT1L results in reduced numbers of progenitors expressing genes including *SoxB1* gene family members. Loss of DOT1L also leads to altered cortical distribution of deep layer neurons that express either TBR1, CTIP2 or SOX5, and less activation of transcriptional programs that are characteristic for upper layer neurons (*Satb2, Pou3f3, Cux2, SoxC* family members). Data from three different mouse models suggest that DOT1L balances transcriptional programs necessary for proper neuronal composition and distribution in the six cortical layers. Furthermore, because loss of DOT1L in the pre-neurogenic phase of development impairs specifically generation of SATB2-expressing upper layer neurons, our data suggest that DOT1L primes upper layer identity in cortical progenitors.

## INTRODUCTION

Modifications of the epigenome, including histone modifications, play a crucial role in neuronal differentiation (1). Dysregulation of specific epigenetic mechanisms have been implicated in neurodevelopmental disorders (2,3). However, only limited description on the impact of individual chromatin modifiers on epigenetic regulation during cortical development is available (4,5). Moreover, the epigenetic mechanisms underlying the spatio-temporal expression of transcription factors (TF) during central nervous system (CNS) development are yet to be elucidated (6,7). During cortical development, gradients of TF activity orchestrate neuronal cell fate commitment (6,8,9). Examples of instructive TF in cortical development are the SRY-box (*Sox*) family members, namely SOX2, SOX4, SOX5 and SOX11 (10–12). Further regulatory TF networks involved in neuronal differentiation and cortical patterning have been extensively described. But many of the molecular mechanisms that control, establish and maintain the hierarchy of TF cascades are still unresolved. In particular, molecular mechanisms that govern cell-intrinsic fate decision toward upper layer (UL) neuronal identity still remain obscure (13). By unknown molecular adaptation processes, progenitors of the cerebral cortex acquire a restrictive developmental potential over time. The cell fate restriction is indicated by the observation that young progenitors can adapt their fate to later developmental programs. In contrast, late progenitors keep their fate information for UL even when transplanted in younger brains (14,15). It is not clear yet at what stage of development an UL fate is established and what might be the underlying molecular mechanism.

Here, we report that activity of the Disruptor of telomeric silencing-like 1 (DOT1L) might prime UL neuronal identity already at the beginning of the neurogenic phase. DOT1L is a histone methyltransferase that mediates specifically histone H3 lysine 79 (H3K79) mono- (me1), di- (me2) and trimethylation (me3), although other enzymes may exist with the same function (16). DOT1L influences different cellular processes such as proliferation, DNA repair, and reprograming in some forms of leukaemia (17). DOT1L is indispensable for early mouse development and promotes proliferation, in particular if stem cells grow under differentiating conditions (18,19). During organ development, DOT1L is implicated in myocardiocyte differentiation, and mainly acts as transcriptional activator (20,21). However, in the forebrain, DOT1L also suppresses transcriptional activation of endoplasmic reticulum (ER) stress programs in primary cortical neurons *in vitro* (22). A growing body of data describes DOT1L function in different cell types, where it affects cell proliferation as well as other properties. But studies that aim to reveal the physiological roles of DOT1L rarely report on mechanisms or target genes that cause the reported phenotypes. Therefore, we aimed to address the function of DOT1L in balancing proliferation and differentiation during CNS development, where *in vivo* target genes are also unknown.

The data presented here show that DOT1L (i) prevents premature cell cycle exit of progenitors, at least in part by affecting asymmetric cell division, and (ii) supports transcriptional programs characteristic for UL cell fate during early cortical development. DOT1L thus primes progenitors for UL gene expression and cell fate before UL neuronal differentiation is thought to occur. Our data suggest that the H3K79me epigenetic modification might provide early-established cell fate information that is able to be transmitted to subsequent progenitor generations.

## MATERIALS AND METHODS

### Mice

Forkhead box G1 (*Foxg1*)-Cre/+ (23), Empty spiracles homeobox 1 (*Emx1*)-Cre/+ (24), and Nestin(*Nes*)-CreER(T2)/R26R-(YFP) (25) animals were mated with floxed *Dot1l*. Animals with the genotype *Foxg1*^cre/+^ or *Emx1*^cre/+^;*Dot1l*^flox/flox^ (cKO) were analyzed in comparison to littermates with *Foxg1*^+/+^ or *Emx1*^+/+^;*Dot1l*^flox/+^ (ctrl). For *Nes-*Cre line pregnant animals were injected with 200μg/g body weight tamoxifen (Tmx) (#T5648, Sigma-Aldrich, USA) and 100 μg/g body weight progesterone (#P0130, Sigma-Aldrich). Non Tmx exposed animals were used as controls. Animal welfare committees of the University of Freiburg and local authorities approved all animal experiments (G12/13, G16/11). Genotyping was performed using primers listed in Supplemental Table S1.

### ChIP, ChIP-seq, and RNA-seq

ChIP-seq for H3K79me2 was performed from E12.5 entire forebrains, which were acutely dissociated into single cells and fixed as described (22). Data from E14.5 time point derived from acutely isolated NPCs from cerebral cortex (22). For H3K4me3 ChIP-seq E14.5 NPC cells were fixed for 5 min at RT with 1% PFA, washed twice with ice-cold PBS and lysed for 10 min on ice with lysis buffer (1% SDS, 10 mM EDTA, 50 mM Tris–HCl pH 8). Antibodies used are listed in Supplemental Table S2. ChIP-seq samples were sequenced on Illumina HiSeq2500 (paired end, multiplexing run, 50 million/reads per sample). For ChIP followed by qRTPCR E14.5 DT cortical tissue was used. This was homogenized, fixed and afterward processed as described above. For RNA-seq, RNA was extracted as described in supplementary methods. The quality of the RNA was assessed with a QIAxcel (#9001941, Qiagen, Germany) and sequenced with an Illumina HiSeq2500. A paired end, multiplexing run was used for sequencing and generated 75 million/reads per sample.

### *In situ* hybridization (ISH), Hematoxylin-Eosin (HE) staining, and immunostainings

ISH, HE staining, and immunostaining of brain tissue and cultured cells was performed as previously described (26,27). For ISH, probes listed in Supplemental Table S1 were applied. Antibodies used are listed in Supplemental Table S2. Information about imaging and quantifications are provided in the supplementary methods.

### *In utero* electroporation (IUE)

IUE was carried out in C57BL/6 (Janvier Labs, Saint Berthevin, France) time-pregnant mice as previously described (28). Briefly, E12.5 pregnant mice were deeply anesthetized with isofluorane, and the uterine horns carrying the embryos were exposed. One lateral ventricle per embryo was injected with 1–2 μl of plasmid DNA (DOT1L-overexpression construct together with pDSV-mRFPnls, or pDSV-mRFPnls alone) at a concentration of 2 μg/μl. Six pulses of 30 V were delivered through the embryonic head. The uterus was repositioned within the abdominal cavity, and after suturing the embryonic development continued normally. At the designated time-points, the embryonic brains were removed and fixed overnight in 4% PFA at 4°C. After extensive rinsing in PBS the brains were processed for immunostaining.

### Bioinformatics of RNA-seq and ChIP-seq

RNA-seq and ChIP-seq data were analyzed on the Galaxy platform (29). RNA-seq FASTQ files were analyzed using following tools: TrimGalore for trimming (30), TopHat2 for read mapping (31,32), HTseq-count for read counting (33) and DESeq2 for differential gene expression analysis (34). ChIP-seq FASTQ files were analyzed using following tools: Bowtie2 for read mapping (35), MACS2 for peak calling (36), DiffBind for differential binding (37) and deepTools2 for in-depth ChIP-seq analysis (38). Detailed analysis steps are provided within the supplemental methods.

### Statistical analysis

Statistical comparisons were performed with GraphPad Prism 6 software. For *in vivo* experiments each n is a different animal. For *in vitro* experiments each n was obtained from a different mESC differentiation. Exemplary data sets for cell numbers and qRTPCRs passed the D’Agostino-Pearson omnibus normality test. Cell numbers within a width of 200 μm of the cortex were normalized to the area in each bin (cell/mm^2^) (Supplementary Figure S2C and D), and compared using an unpaired, two-tailed Student's *t*-test (equal variances) or Welch's *t*-test (unequal variances). For qRTPCRs data analysis, ΔCt values normalized to the house keeping gene *Gapdh* were compared using an unpaired, two-tailed Student's *t*-test (equal variances) or Welch's *t*-test (unequal variances). For data presentation, ΔΔCt values are expressed as log_2_(fold change) and shown as mean ± SEM. These values were calculated from three technical replicates from each independent *n*. The error propagation formula was used to assess correct SEM values. For statistical analyses of γ-TUBULIN immunostainings, Fisher's exact test on a 2 × 2 contingency table with small sample size was used.

## RESULTS

### *Dot1l*-deficiency impairs cortical development

**Figure 1.**
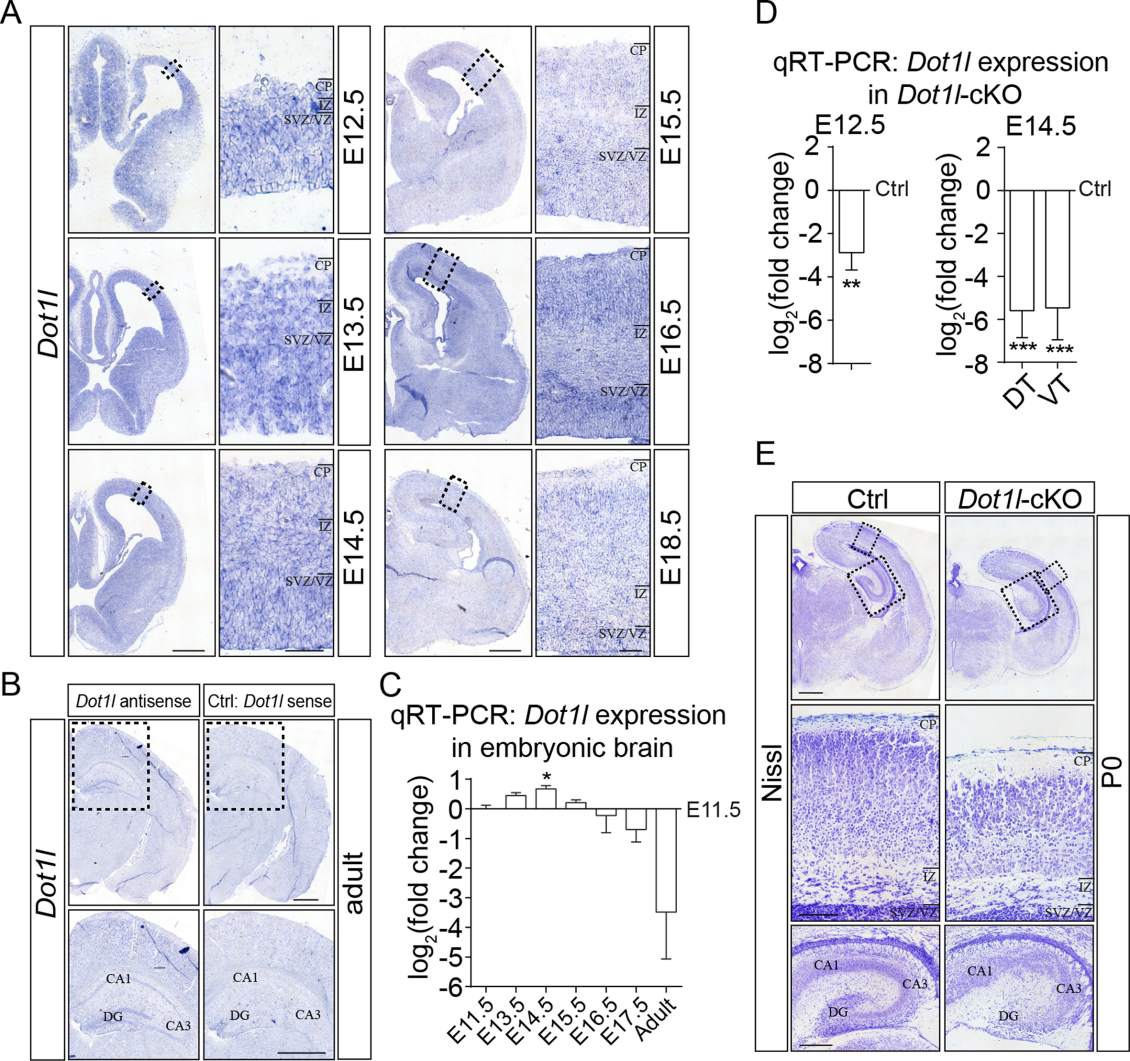
*Dot1l* is expressed in the developing mouse cerebral cortex and prevents microcephaly. (**A, B**) *In situ* hybridization (ISH) of cortical sections using *Dot1l-*antisense probe at different developmental time points (E12.5-E18.5) reveals expression of *Dot1l* during mouse brain development (A), and in adult brains (B). Annotations: Cortical plate (CP), intermediate zone (IZ), subventricular/ventricular zone (SVZ/VZ), cornu ammonis (CA), dentate gyrus (DG). Scale bars: overview 500 μm, magnification 50 μm. (**C**) qRTPCR analysis of *Dot1l* expression at different embryonic stages (E13.5-E17.5, *n* = 3) and in adult (*n* = 4) cortical tissue normalized to E11.5. (**D**) qRTPCR analysis of *Dot1l* expression in *Foxg1-*Cre *Dot1l-*cKO animals normalized to values of control (ctrl) animals (*n* = 6) at E12.5 in the entire forebrain and E14.5 in dorsal (DT) and ventral telencephalon (VT). (**E**) Nissl stained sections of P0 ctrl and *Foxg1-*Cre *Dot1l-*cKO brains. Black squares in upper panels represent higher magnification images depicted below. Scale bars: 500 μm. Data of qRTPCRs are presented as mean ± SEM. *P*-values were calculated using unpaired, two tailed Student's *t*-test: *P < 0.05, **P < 0.01, ***P < 0.001.

In the developing cerebral cortex, *Dot1l* is expressed in the progenitor zone and cortical plate between E11.5 and the adult stage (Figure 1A, B). In quantitative real-time PCR (qRTPCR), *Dot1l* was significantly elevated at the early neurogenesis stage E14.5 compared to E11.5 (Figure 1C). To elucidate DOT1L function in the cerebral cortex, we generated a DOT1L conditional knockout mouse (*Dot1l-*cKO) using a *Foxg1*-Cre driver line (23). *Dot1l-*cKO mice displayed a significant depletion of *Dot1l* mRNA in the telencephalon at E12.5 and E14.5 (Figure 1D). *Dot1l-*cKO animals died within minutes after birth. Histological characterization at P0 showed that *Dot1l-*cKO animals had microcephaly affecting ventral and dorsal structures (Figure 1E). The cortical plate of mutant brains was generally thinner compared to controls and cells appeared less densely packed. In addition, the hippocampus was disorganized with fewer cells in the dentate gyrus at P0 (Figure 1E). Heterozygote *Foxg1-*Cre mice are reported in some studies to have a mild cortical phenotype (39,40). We therefore also used an *Emx1-*Cre driver line (24) to delete *Dot1l*. Nissl staining of *Emx1*-Cre induced *Dot1l*-cKO at P0 showed that microcephaly was less obvious in these mutant brains compared to *Foxg1*-Cre mediated deletion of DOT1L. However, the cellular composition of the cortical plate seemed to be different between mutant and control brains (Supplementary Figure S1A). The hippocampus of *Emx1*-Cre induced *Dot1l*-cKO was also disorganized, especially in the dentate gyrus. In all, we observed a comparable phenotype between *Foxg1*-Cre and *Emx1*-Cre. But as the microcephalic phenotype was stronger in *Foxg1*-Cre *Dot1l-*cKO, we continued with further phenotypic characterizations in this mouse line.

### *Dot1l*-deficiency reduces the number of progenitors, neurogenesis as well as distribution of neuronal subtypes

**Figure 2.**
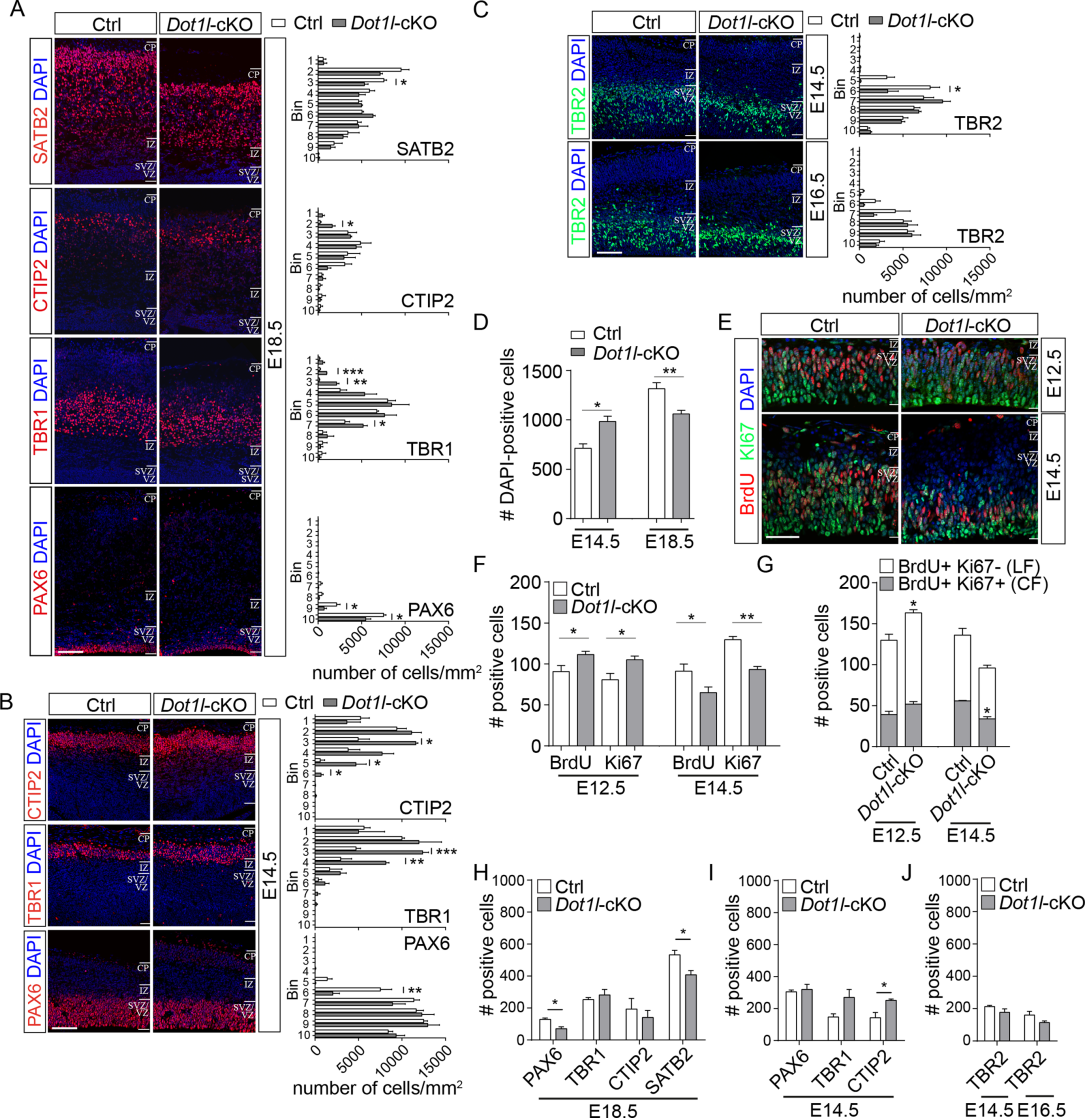
*Dot1l*-cKO leads to decreased numbers of progenitors and UL neurons, as well as altered distribution of DL neurons. (**A**) Left: Immunostainings of E18.5 forebrain sections for PAX6, TBR1, CITP2, and SATB2 in red, DAPI in blue. Annotations: Cortical plate (CP), intermediate zone (IZ), subventricular/ventricular zone (SVZ/VZ), Scale bar: 100 μm. Right: Quantification of number of stained cells/mm^2^ within each individual bin out of 10 bins spanning the entire cortex from VZ (bin 10) to the marginal zone (bin 1) in a width of 200 μm (PAX6 *n* = 3, TBR1 *n* = 3, CTIP2 *n* = 3(ctrl)/4(*Dot1l*-cKO), SATB2 *n* = 3) for ctrl and *Foxg1*-Cre*Dot1l*-cKO. (**B**) Immunostainings of E14.5 forebrain sections for PAX6, TBR1, and CTIP2 (left) and quantification of total number of stained cells/mm^2^ (PAX6 *n* = 4(ctrl)/6(*Dot1l*-cKO),TBR1 *n* = 4, CTIP2 *n* = 4) in 10 bins (right). Scale bar: 100 μm. (**C**) Immunostainings (left) and quantification (right) of TBR2-positive cells at E14.5 and E16.5 (*n* = 3). (**D**) Total number of DAPI-stained nuclei obtained from forebrain sections at E14.5 and E18.5 in a width of 200 μm (*n* = 3(ctrl)/4(*Dot1l*-cKO)). (**E**) Representative images of immunostainings of E12.5 and E14.5 forebrain sections for BrdU (red), Ki67 (green), and DAPI (blue). Scale bar: 200 μm. (**F**) Quantification of number of BrdU and Ki67 stained cells in a width of 200 μm (*n* = 4). (**G**) Quantification of BrdU+/Ki67- cells, representing the leaving fraction (LF), and BrdU+/Ki67+ cells representing the cycling fraction (CF) in 200 μm width of the cortex at E12.5 and E14.5. (**H**) Total numbers of PAX6, TBR1, CTIP2, or SATB2-positive cells at E18.5 in 200 μm width of the cortex, as determined from the sum of positive cells for the respective markers counted in B. (**I**) PAX6, TBR1, or CTIP2 positive cells at E14.5 in 200 μm width of the cortex, as determined from the sum of positive cells for the respective markers counted in C. (**J**) TBR2-positive cells at E14.5 and E16.5 in 200 μm width of the cortical plate as determined from the sum of positive cells for the different time points counted in D. All data are from ctrl and *Foxg1*-Cre*Dot1l*-cKO and represented as mean ± SEM. *P*-values were calculated using unpaired, two tailed Student's *t*-test: *P < 0.05, **P < 0.01, ***P < 0.001.

Immunostaining experiments at E18.5 revealed fewer Paired box 6 (PAX6)-positive progenitors in the *Dot1l-*cKO compared to controls (Figure 2A). Deep layer (DL) neurons marked by T-box brain 1 (TBR1) and B cell leukemia/lymphoma 11B (BCL11B/CTIP2) (41) were significantly increased in the *Dot1l-*cKO brains (Figure 2A). In contrast, upper layer (UL) neurons, identified by Special AT-rich sequence binding protein 2 (SATB2) (42), significantly decreased upon DOT1L-deficiency. At E14.5, *Dot1l-*deficient mice exhibited similar changes with decreased numbers of PAX6-, and increased numbers of TBR1- and CTIP2-expressing cells compared to controls (Figure 2B). TBR2-expressing intermediate progenitor cells (IPCs) also decreased at E14.5 (Figure 2C). *In situ* hybridization (ISH) studies suggested altered expression of Reelin (*Reln*) (43) (Supplementary Figure S2A), and a thinner expression domain of LIM homeobox protein 2 (*Lhx2*) in the knockout as compared to control mice (Supplementary Figure S2B).

Together, these alterations of marker gene expression suggested that *Dot1l-*cKO reduced neural progenitor cells and promoted their premature differentiation into DL neurons, at the expense of UL neurons. To compare quantitated cell numbers in the microcephalic and control cortices, we divided the cortical plate into 10 bins and determined cell numbers in each bin. This approach allowed us to normalize to the variable height of the cortices in the different genotypes (Supplementary Figure S2C, D). However, the significant changes observed within specific bins (Figure 2A–C) did not resolve whether they resulted from differences in generation or rather through aberrant distribution of the mature neurons in the cortical plate. To address the question of differences in neurogenesis or distribution of cells, we first quantified the total number of DAPI-positive cells in the cortical plate at E14.5 and E18.5. This revealed increased numbers of cells at E14.5 and decreased numbers at E18.5 in DOT1L-deficient mice as compared to controls (Figure 2D). These findings suggested that increased cell proliferation in the early neurogenic phase, premature progenitor differentiation and reduction of the progenitor pool could account for the phenotype of the *Dot1l-*cKO mice. To test for a potential increase in cell proliferation in early developmental stages upon *Dot1l-*cKO, we labeled S-phase cells by a 1h BrdU pulse, and quantified numbers of BrdU- and Ki67-positive cells at E12.5 and E14.5 (Figure 2E, F). Consistent with our prediction, *Dot1l-*cKO mice had significantly increased cell proliferation at E12.5 compared to controls, and decreased proliferation at E14.5 (Figure 2F). In addition, a significant higher fraction of *Dot1l*-cKO cells left the cell cycle (BrdU-labeled only, leaving fraction (LF)) at E12.5 compared to control cells. At E14.5 the number of proliferating cells and the cycling fraction (BrdU- and Ki67-positive, cycling fraction (CF)) decreased in mutant compared to control animals (Figure 2G). Thus, these BrdU pulse chasing experiments supported the finding of increased proliferation in the early neurogenic phase, premature progenitor differentiation and reduction of the progenitor pool in *Dot1l-*cKO mice.

Next, we quantified the total numbers of PAX6-, TBR2-, TBR1-, CTIP2- and SATB2-expressing cells. Loss of DOT1L reduced the total number of PAX6- and SATB2-positive cells at E18.5 compared to controls, whereas total numbers of TBR1- and CTIP2-positive neurons did not change (Figure 2H). At E14.5, the total number of PAX6-positive cells remained constant, but we observed more TBR1- and CTIP2-expressing cells in the *Dot1l*-cKO compared to controls (Figure 2I). The total number of TBR2-positive cells was constant between genotypes (Figure 2J). In summary, *Dot1l-*cKO decreased the generation of PAX6-positive progenitors and SATB2-expressing UL neurons compared to controls. Loss of DOT1L caused premature appearance of more TBR1- and CTIP2-expressing cells. The total number of these DL neurons was similar between the genotypes at the later embryonic stage E18.5. The quantitative differences of TBR1- and CTIP2-positive neurons within different bins of the cortex between DOT1L-deficient and control animals thus reflected altered distribution of these neuronal subtypes at E18.5. Neither increased apoptosis and/or expression of ER stress genes (22) were observed in *Dot1l-*cKO compared to controls to account for the altered numbers of cells (Supplementary Figure S3A-E).

We next assessed whether *Emx1-*Cre driven *Dot1l-*cKO mice had comparable phenotypic alterations to using *Foxg1*-Cre. Analysis by immunostainings followed by quantification of layer markers at E18.5 revealed a comparable pattern of *Emx1-*Cre to *Foxg1*-Cre mediated derivation of *Dot1l-*cKO mice (Supplementary Figure S4A). In both cases, deletion of DOT1L either through *Emx1-*Cre or *Foxg1*-Cre reduced the numbers of PAX6-, TBR2-, and SATB2-cells, and led to altered distribution of TBR1- and CTIP2-neurons (Supplementary Figure S4A, B).

In all, we concluded that the *Dot1l-*cKO on one hand increased cell proliferation in the early neurogenic phase, and prematurely reduced the progenitor pool through neuronal differentiation. On the other hand, we observed aberrant cell positioning of DL neurons and decreased numbers of SATB2-positive UL neurons in DOT1L-deficient compared to control brains.

### H3K79me2 marks genes expressed in progenitors and UL neurons

Genome-wide sequencing served as a baseline to explore the molecular nature of the phenotypic alterations occurring in *Dot1l-*cKO mice. We analyzed the pattern of H3K79me2 deposited on the chromatin in wild-type animals using chromatin immunoprecipitation (ChIP) followed by genome-wide sequencing (ChIP-seq) at E12.5 and E14.5. Additionally, at E14.5 we performed ChIP-seq analysis of H3K4me3 that marks active transcription (44), as well as genome-wide sequencing of mRNA (RNA-seq) of *Dot1l*-cKO and controls in the dorsal telencephalon.

**Figure 3.**
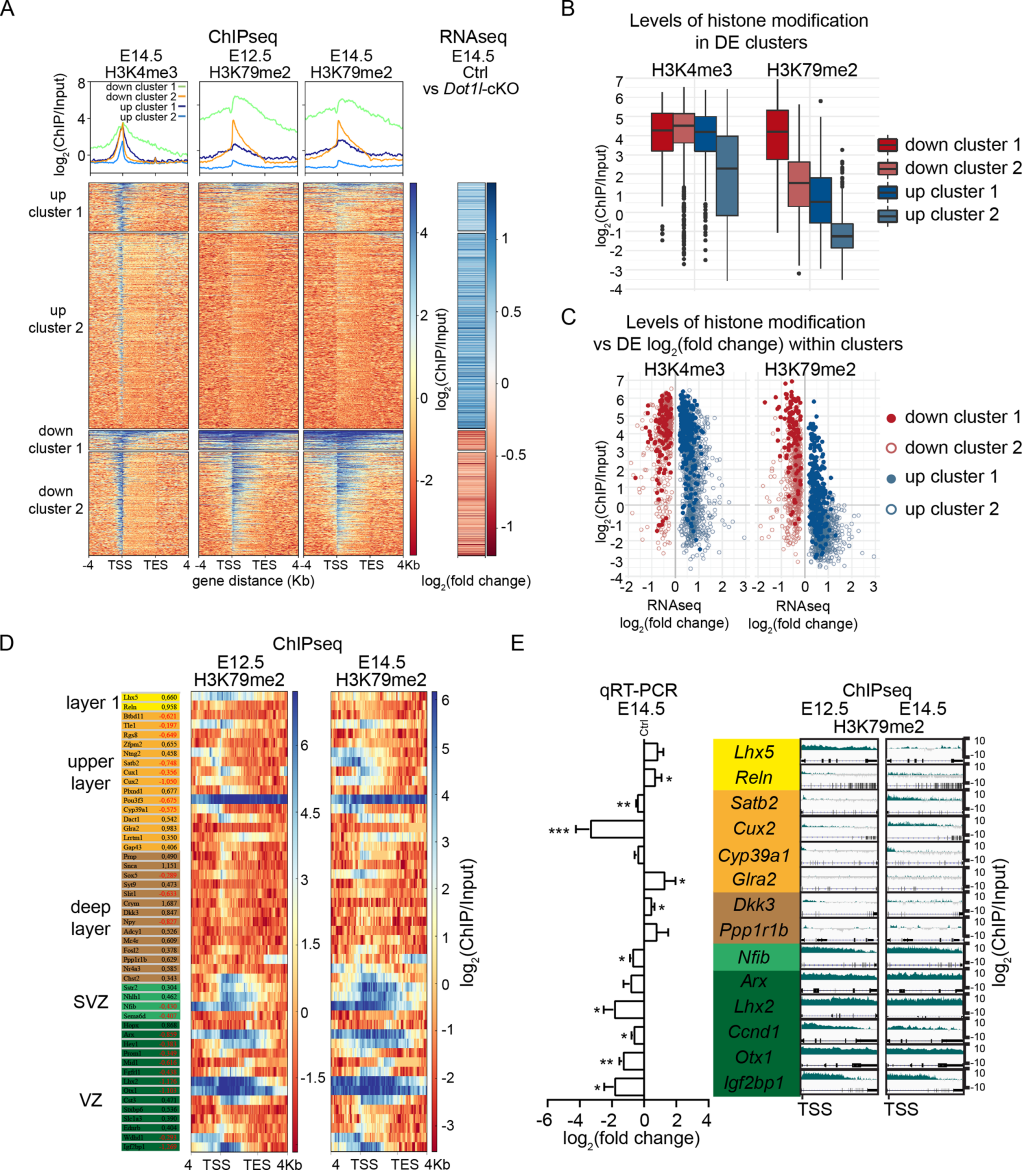
Genes expressed in VZ, SVZ, and UL of the cortical plate are enriched with H3K79me2 and their expression decreased significantly upon *Dot1l-*cKO. (**A**) Lower panels: Heatmaps of enriched (blue) and non-enriched (red) read counts for H3K4me3 (E14.5) and H3K79me2 (E12.5 and E14.5) along the gene body (scaled to 4 kb) including the flanking regions +/-4 kb after ChIP-seq from wild type forebrains, arranged in four K-means clusters according to H3K79me2 levels at E14.5. All DE genes upon *Foxg1-*Cre *Dot1l-*cKO at E14.5 including their expression levels according to RNA-seq in log_2_(fold change) are displayed (right panel); blue: increased, red: decreased expression. Upper panels: distribution of ChIP enrichment as log_2_(ChIP/Input) for the individual histone marks over all genes and subdivided according to the four K-means clusters, indicating highest enrichment around the TSS and in down cluster 1 (green), and lower enrichment toward the TES and in down cluster 2 (orange), up cluster 1 (black) and 2 (blue). (**B**) log_2_(ChIP/Input) average at the TSS ±250 bp of H3K4me3 and H3K79me2 in the four clusters (down cluster 1, down cluster 2, up cluster 1, up cluster 2) of differentially expressed genes. (**C**) Correlation analysis of H3K4me3 and H3K79me3 levels (log_2_ (ChIP/Input) versus expression of genes (log_2_(fold changes)), represented according to K-means clusters. (**D**) Arrangement of DE genes, expression of which is confined to VZ (dark green), SVZ (light green), deep layers (brown), upper layers (orange), and layer 1 (yellow). Given are the gene names, fold changes in RNA-seq data compared to ctrl (left panel), and heatmaps along the gene body (+/-4 kb, including TSS and TES) of H3K79me2 pattern (blue: strong enrichment, red: no enrichment) for the respective genes at E12.5 and E14.5 in wild type animals (right panel). (**E**) qRTPCR validation of selected layer-specific genes, color-coded as in D (left panel) (*Otx1, Ccnd1* and *Cyp39a1n* = 3; *Lhx5, Lhx2* and *Igfbp1n* = 4; *Nfibn* = 5, *Arx, Ppp1r1b, Dkk3, Glra2, Cux2* and *Relnn* = 6; Satb2 *n* = 8). High resolution representation of H3K79me2 distribution at the respective genes from TSS to TES in E12.5 and E14.5 wild type dorsal telencephalon represented as log_2_(ChIP/input) (right panel), indicating stronger enrichment in progenitor and UL compared to DL expressed genes. All qRTPCR data are represented as mean ± SEM. *P*-values were calculated using unpaired, two tailed Student's *t*-test: *P < 0.05, **P < 0.01, ***P < 0.001.

RNA-seq revealed 1976 significantly (adjusted *P*-value ≤ 0.05) differentially expressed (DE) genes upon *Dot1l-*cKO. We investigated the distribution of the H3K4me3 and H3K79me2 marks within the gene bodies (spanning from the transcriptional start site (TSS) to the transcriptional end site (TES)) and adjacent ±4 kb of the 1976 DE genes (Figure 3A, lower panel). Almost all DE genes were marked with H3K4me3, whether they showed decreased or increased expression upon *Dot1l-*cKO compared to controls (Figure 3A). To reveal any correlations between H3K79me2 and transcriptional alterations of the 1976 target genes, we used K-means clustering with a preset of four clusters. Two clusters contained genes with increased expression, of which *up cluster 1* contained 260 genes with higher levels of H3K79me2, compared to *up cluster 2* with 1054 genes (Figure 3B). The other two clusters contained genes with decreased expression. 104 genes in *down cluster 1* were transcriptionally decreased and had substantial H3K79me2 modification up- and downstream of the TSS; 558 *down cluster 2* genes had decreased expression and H3K79me2 mainly downstream of the TSS (Figure 3A, upper and lower panel). Quantitative analyses of H3K4me3 and H3K79me2 levels between the four clusters showed similar distribution of values for H3K4me3 in all clusters except for *up cluster 2*, which had slightly lower levels compared to the other clusters (Figure 3B). In contrast, H3K79me2 levels were higher for genes with decreased transcription upon *Dot1l*-cKO (*down cluster 1* and *2*, Figure 3B), compared to the genes with increased transcription. As shown in Figure 3C, most of the transcriptional alterations upon *Dot1l-*cKO were moderate, and DE genes with high enrichment for H3K79me2 both transcriptionally increased or decreased. Those genes that did not change upon *Dot1l-*cKO overall had a lower H3K79me2 coverage compared to the DE genes (Supplementary Figure S5A). Together this data indicated variability of H3K79me2 patterns. On one hand this variability was observed with regard to the location within the genomic region, whereby H3K79me2 was either enriched at the TSS or it was deposited over larger regions, including entire gene bodies. On the other hand, H3K79me2 was present at genes that transcriptionally increased or decreased upon DOT1L-deletion compared to controls.

We concluded that for identification of DOT1L target genes detailed, locus-specific analyses of H3K79me2 patterns together with expression changes upon DOT1L-deficiency might be necessary. Our further analyses therefore contain both information, H3K79me2 pattern and gene expression. We hypothesized from this data set, that direct DOT1L targets might be enriched among genes which were highly marked by H3K79me2 and decreased upon DOT1L-deficiency. Thus, our further experimentation aimed (1) to identify specific DOT1L target genes that might be causing the observed phenotype and (2) to test this hypothesis.

With the aim to identify specific DOT1L target genes, we considered a potential contribution of the reduced *Foxg1* expression (caused by the *Cre* knockin) could lead to the transcriptional alterations observed in *Dot1l-*cKO (39). Therefore, we performed RNA-seq of heterozygote *Foxg1-*Cre forebrains and intersected the 402 significantly DE genes with the 1976 DE genes revealed from DOT1L-deficient forebrains (Supplementary Figure S5B). We identified 132 shared genes, 70 of which did not carry H3K79me2 (Supplementary Figure S5C), and excluded these 132 DE transcripts from our further analyses.

The phenotype of the developing cortical plate suggested that the *Dot1l-*cKO affected both progenitor proliferation and neuronal differentiation. We therefore analyzed whether DOT1L-mediated H3K79me2 modification correlated with expression changes of genes normally expressed in the ventricular zone (VZ), subventricular zone (SVZ), DL, UL, and layer 1 (the classification of these genes was extracted from various publications, which are summarized in Supplemental Table S3). The corresponding heatmap of the H3K79me2 ChIP-seq profile of the selected transcripts in the different cellular subtypes revealed that out of 18 selected genes expressed in VZ and SVZ progenitors, 16 carried H3K79me2 at E12.5, and 18 at E14.5, respectively. Eleven of these 18 transcripts decreased upon *Dot1l-*cKO (Figure 3D). Of 14 selected genes expressed in DL neurons, 9 had few or no H3K79me2 marks at E12.5, and 7 at E14.5, respectively, and 11 of these DL transcripts increased upon loss of DOT1L. 15 genes were selected because their expression has been identified in UL neurons. Thirteen out of 15 genes were already marked with H3K79me2 at E12.5, and 11 at E14.5. 8 of this set of candidate genes decreased in *Dot1l-*cKO forebrains compared to controls (Figure 3D). qRTPCR of a subset of these selected candidates confirmed the transcriptional changes observed by RNA-seq (Figure 3E, left panel).

It is of note that the H3K79me2 pattern differed substantially between the selected genes (Figure 3D). We therefore used locus-specific high resolution analyses to identify gene-specific H3K79me2 enrichments, which we hypothesized serve best to identify direct DOT1L target genes. We correlated expression levels and such H3K79me2 profiles in progenitors and neurons. This analysis indicated higher enrichment of the histone modification in progenitors compared to neurons (Figure 3E, right panel). Among neuronal genes, those with DL expression (Dickkopf WNT signaling pathway inhibitor 3 (*Dkk3*), Protein phosphatase 1 regulatory inhibitor subunit 1B *(Ppp1r1b*)) had lower H3K79me2 enrichment than UL expressed genes (*Satb2*, Cut like homeobox 2 *(Cux2*)). Taken together, ChIP-, RNA-seq, and qRTPCR data supported our hypothesis that direct DOT1L targets are most likely, but not exclusively, identified by high levels of H3K79me2 and decreased expression upon *Dot1l*-cKO. Among genes expressed by specific cell populations during cortical development, we identified Insulin like growth factor 2 mRNA binding protein 1 (*Igf2bp1*), Orthodenticle homeobox 1 *(Otx1*), Cyclin D1 (*Ccnd1*), *Lhx2* and Nuclear factor I B (*Nfib*) as novel targets in progenitors, and *Satb2* as well as *Cux2* as UL targets. From transcripts expressed by DL neurons, only *Sox5* classified as DOT1L target under our selection criteria. In addition, these data suggested to us that DOT1L might infer neuronal cell fate information because we identified target genes such as *Satb2* and *Cux2*. These latter genes are expressed by late born neurons, but were marked with H3K79me2 already at a developmental stage dominated by proliferative events, i.e. E12.5.

### DOT1L and H3K79me2 activate transcription of HMG-domain transcription factors

**Figure 4.**
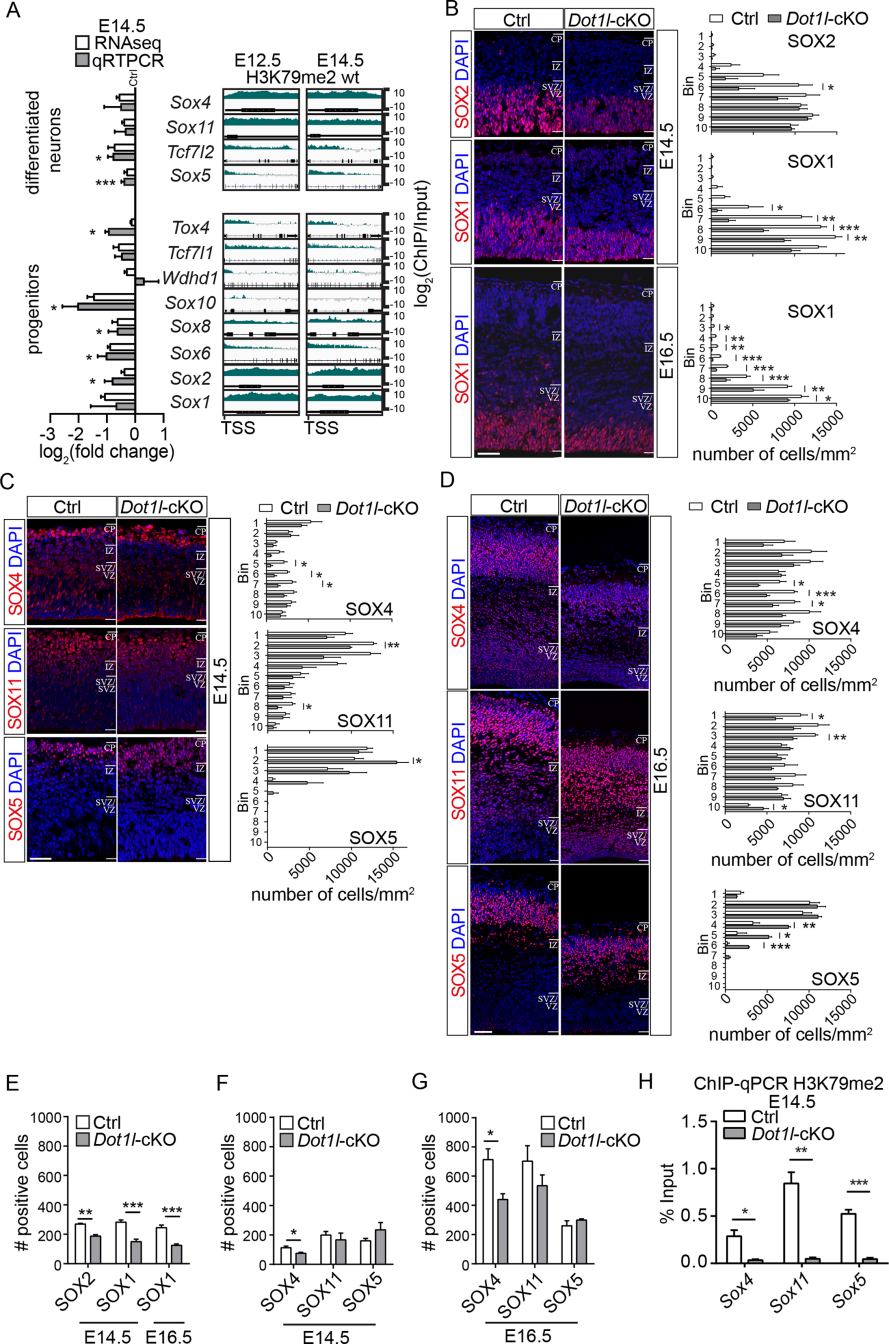
*Sox* family members are direct targets of DOT1L during cortical development *in vivo* and *in vitro*. (**A**) Left panel: Differences in expression levels of *Sox* family members in log_2_(fold change) in *Foxg1-*Cre *Dot1l-*cKO compared to ctrl in RNA-seq (*n* = 3) (white bars) and qRTPCR validations (gray bars, *n* = 6). Right panels: high resolution representation of H3K79me2 enrichment in log_2_(ChIP/Input) at E12.5 and E14.5 of the indicated genes, which are subdivided according to expression mainly in progenitors or differentiated neurons. (**B**) Representative images (left panels) and quantifications (cells/mm^2^ in 10 bins, right panels) of immunostainings of SOX1 (*n* = 4) and SOX2 (*n* = 3) at E14.5, and SOX1 (*n* = 3) at E16.5. Scale bar: 100 μm. (**C**) Representation as in B for SOX4 (*n* = 3), SOX5 (*n* = 4), and SOX11 (*n* = 3) at E14.5. (**D**) Representation as in B for SOX4 (*n* = 4), SOX5 (*n* = 3), and SOX11 (*n* = 4) at E16.5. (**E**) Total numbers of SOX1- and SOX2-positive cells at E14.5 and E16.5 in 200 μm width of the cortex, as determined from the sum of positive cells for the respective markers counted in B for ctrl and *Foxg1*-Cre*Dot1l*-cKO. (**F**) Total numbers SOX4-, SOX11-, and SOX5-positive cells at E14.5 in 200 μm width of the cortex, as determined from the sum of positive cells for the respective markers counted in C. (**G**) Total numbers of SOX4-, SOX11- and SOX5-positive cells at E16.5 in 200 μm width of the cortex, as determined from the sum of positive cells for the respective markers counted in D. (**H**) ChIP-qPCR analysis of H3K79me2 levels (% Input) of E14.5 ctrl and *Foxg1-*Cre *Dot1l*-cKO at the promotor region of *Sox4, Sox5*, and *Sox11* (*n* = 4). All data are from ctrl and *Foxg1*-Cre*Dot1l*-cKO and represented as mean ± SEM. *P*-values were calculated using unpaired, two tailed Student's *t*-test: *P < 0.05, **P < 0.01, ***P < 0.001.

We aimed to identify further DOT1L target genes, which fulfilled the selection criteria of decreased transcription and substantial H3K79me2 at E14.5. We observed enrichment of many HMG-domain TF-family members, namely *Sox1, Sox2, Sox4, Sox5, Sox6, Sox8, Sox10, Sox11*, Transcription factor 7 like 1 *(Tcf7l1), Tcf7l2*, WD repeat and HMG-box DNA binding protein 1 *(Wdhd1*) and Thymocyte selection-associated HMG box (*Tox1*). With the exception of the oligodendrocyte-expressed *Sox10* (45), all HMG TF were marked with H3K79me2 at E12.5 and E14.5 (Figure 4A). qRTPCR verified 7 out of 12 HMG TF with significantly decreased expression upon *Dot1l* deletion *in vivo* (Figure 4A).

The activating effect of DOT1L on HMG TF transcription was not restricted to progenitors, but was also evident for family members active in differentiated neurons (Figure 4A), again indicating that DOT1L might act beyond cell proliferation on neuronal differentiation. Immunostaining analysis for SOX1 and SOX2 and confirmed decreased expression in DOT1L-deficient mouse forebrains at E14.5 and E16.5 in the VZ compared to controls (Figure 4B). As the total number of SOX1- and SOX2-positive cells decreased, we concluded that DOT1L function affects the number of these progenitors (Figure 4E). SOX4, SOX5, and SOX11 are expressed in differentiated neurons. SOX5 is expressed in DL neurons, and we quantified significantly increased numbers of SOX5-positive cells in *Dot1l*-deficient forebrains at E14.5 (Figure 4C) and E16.5 (Figure 4D) compared to controls. In contrast, SOX4 and SOX11, which are expressed in DL and UL neurons, significantly decreased in *Dot1l-*cKO (Figure 4C, D). While loss of DOT1L decreased generation of SOX4-positive cells, as revealed by the reduced total number of SOX4-positive cells compared to wild type animals, numbers of SOX5- and SOX11-neurons did not change (Figure 4F, G). This indicated that DOT1L-deficiency most likely affected distribution of SOX5- and SOX11-expressing neurons. qRTPCR after H3K79me2 ChIP revealed less enrichment of marked chromatin in E14.5 *Dot1l-*cKO forebrains compared to controls around the TSS of SOX4, 11 and 5 (Figure 4H), suggesting a direct effect of DOT1L on expression of these neuronal genes.

This data supported the view that DOT1L and H3K79me2 (i) directly regulated HMG TF expression *in vivo*, (ii) decreased the number of progenitors, and (iii) impacted on the generation and distribution of cortical plate neurons, depending on their respective subtype.

### DOT1L-deficiency decreases transcription of genes affecting the cell cycle and the division mode

We used pathway analyses of DE genes upon *Dot1l* deletion to identify further *in vivo* cortical DOT1L target genes. These analyses indicated enrichment for cell cycle-related genes (Supplementary Figure S6A). Fifteen potential DOT1L target genes implicated in cell cycle control carried H3K79me2 modifications and mainly affect G1/S phase. Cyclin D1 (*Ccnd1*) and E2F transcription factor (*E2f7*), which are important for G1/S transition (46), were the only two genes from this set of selected genes with significantly altered expression as revealed using qRTPCR analyses of control and *Dot1l-*cKO dorsal forebrains (Supplementary Figure S6B).

**Figure 5.**
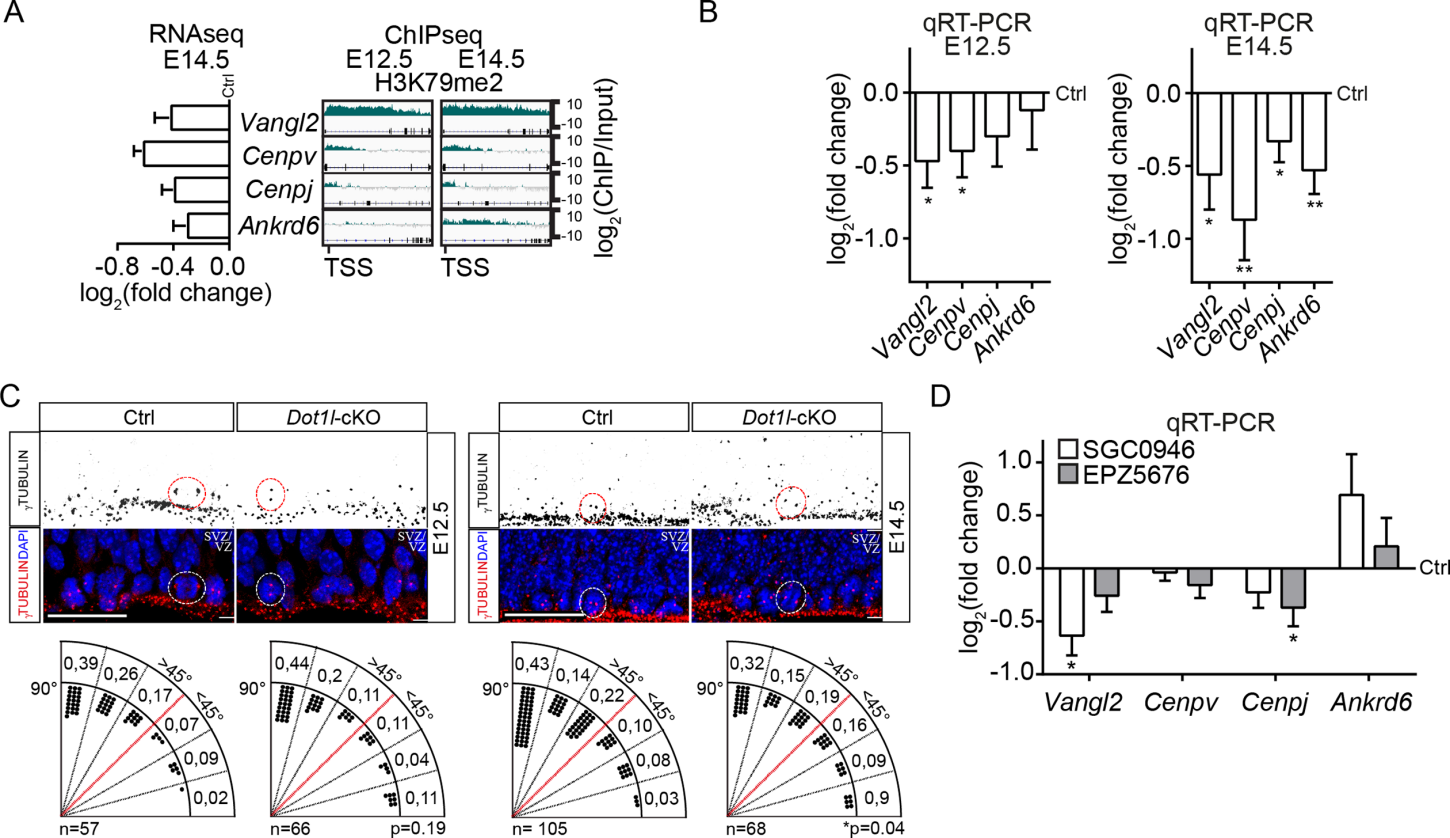
DOT1L induces transcription of genes implicated in asymmetric cell division. (**A**) RNA-seq expression data (*Foxg1*-Cre *Dot1l*-cKO vs ctrl, left panel) and high resolution representation of H3K79me2 distribution along the respective gene in wild type forebrains in log_2_(ChIP/input) at E12.5 and E14.5 of *Vangl2, Cenpv, Cenpj* and *Ankrd6*. (**B**) qRTPCR validation of the expression levels of the genes shown in A in *Foxg1*-Cre*Dot1l*-cKO compared to ctrl at E12.5 and E14.5 (*n* = 6). (**C**) Representative immunostainings for γ-TUBULIN (red) and DAPI (blue) of E12.5 and E14.5 VZ of ctrl and *Foxg1*-Cre *Dot1l*-cKO (middle panels). For better visualization of the γ-TUBULIN staining, the red channel was split and inverted (upper panel). Cells with different orientation of the cleavage planes are depicted exemplarily with red circles. Quantification of the number of cells (represented as black dots) that presented different angles of the cleavage plane in respect to the ventricle surface within increments of 15° is shown in the lower panel for ctrl (*n* = 57 at E12.5, *n* = 105 at E14.5) and *Foxg1*-Cre*Dot1l*-cKO (*n* = 66 at E12.5, *n* = 68 at E14.5). Statistical comparison between *Dot1l*-cKO vs ctrl of all dividing cells with cleavage planes below 45° or above 45° was performed with one-tailed Fisher's exact test at E12.5 or E14.5. *P-*values are shown below the quantifications: *P < 0.05. Scale bars: 25 μm. (**D**) qRTPCR analysis for *Vangl2, Cenpv, Cenpj* and *Ankrd6* in neural progenitor cells differentiated from mESCs after pharmacological inhibition of DOT1L for 48h with two different inhibitors, SGC0946 (white bars) and EPZ5676 (gray bars) compared to DMSO treated ctrl cells (*n* = 4). All data are presented as mean ± SEM. *P*-values were calculated using unpaired, two tailed Student's *t*-test: *P < 0.05, **P < 0.01.

Despite lower numbers of cells in S-phase at E14.5, *Dot1l* deletion did not result in significantly decreased numbers of M-phase cells, as determined by Histone H3 serine 10 phosphorylation (H3S10ph) immunostaining (Supplementary Figure S6C). This seemingly contradicting finding might indicate an altered M-phase. We hypothesized that at E14.5, fewer cells entered S-phase in the *Dot1l*-cKO mice (Figure 2F) and that these cells accumulated in M-phase, which resulted in comparable numbers to control brains with an unperturbed cell cycle. To test this hypothesis, we analyzed genes that are known to influence specifically M-phase with regard to (i) occurrence of H3K79me2 as indicator of direct DOT1L effects, and (ii) decreased gene expression in the RNA-seq data. In support of our hypothesis, ChIP-seq data revealed Vang-like 2 (*Vangl2*), and Centromere protein v *(Cenpv*) and j (*Cenpj*), which carried H3K79me2 modifications at E12.5 and E14.5, and Ankyrin repeat domain 6 (*Ankrd6*), which gained H3K79me2 at E14.5 (Figure 5A). These genes influence M-phase progression and prevent premature progenitor differentiation through asymmetric cell division by maintaining a horizontal cleavage plane (47–49). RNA-seq and qRTPCR validation showed that *Vangl2* and *Cenpv* expression significantly decreased upon *Dot1l* deletion at E12.5, and all four genes decreased in expression compared to controls at E14.5 (Figure 5A, B).

We quantified the number of cells in the VZ with angles of the cleavage plane between 0° and 90° in 15° increments at E12.5 and E14.5 after γ-TUBULIN immunostaining. Loss of DOT1L resulted in significantly more cells with a cleavage plane parallel to the ventricular surface at E14.5, which is a hallmark of asymmetric division (Figure 5C). To ascertain that DOT1L activity impacts on transcription of *Vangl2, Cenpv, Cenpj*, and *Ankrd6*, we differentiated mouse embryonic stem cells (mESC) into radial glial-like cells (RGLC) *in vitro* (50). Interference with DOT1L activity by applying the specific inhibitors SGC0946 or EPZ5676 to RGLC for 48h led to significant lower levels of H3K79me2 modification compared to DMSO treated controls (Supplementary Figure S6D, E). DOT1L-inhibited RGLC expressed significantly less *Vangl2* and *Cenpj* compared to controls (Figure 5D). Taken together, the data showed that DOT1L activity promoted transcription of genes implicated in asymmetric cell division *in vivo* and *in vitro*.

### DOT1L activates transcriptional programs governing UL neuronal cell fate

**Figure 6.**
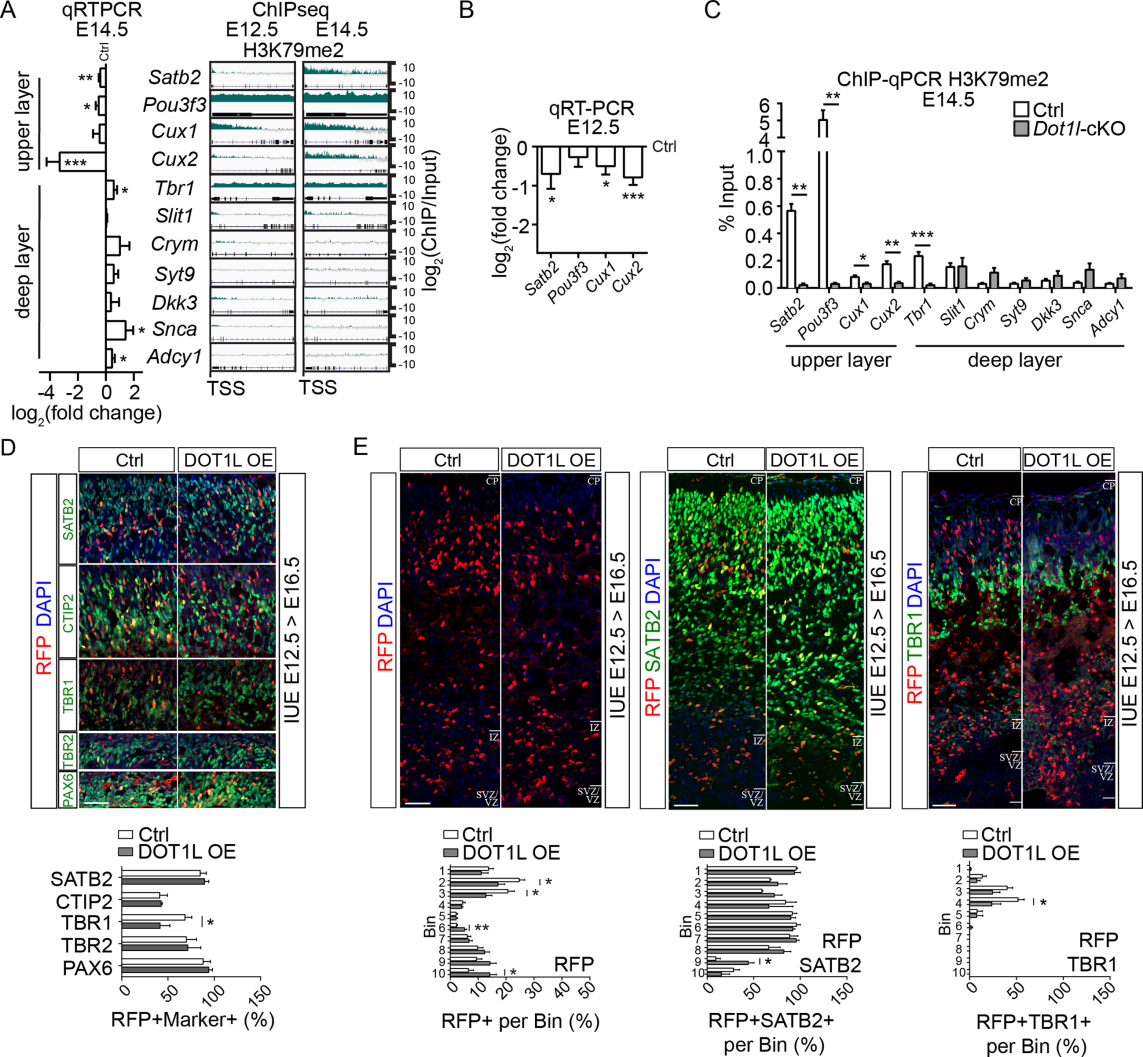
DOT1L activates transcriptional programs governing UL neuronal cell fate. (**A**) qRTPCR expression analysis of genes expressed in UL cortical neurons in *Foxg1*-Cre *Dot1l*-cKO compared to ctrl at E14.5 (*n* = 6, left panel) and high resolution representation of H3K79me2 distribution from TSS over the respective gene body in wild type forebrains in log_2_(ChIP/input) of genes that are expressed in UL or DL cortical neurons at E12.5 and E14.5. (**B**) qRTPCR expression analysis of genes expressed in UL cortical neurons in *Foxg1*-Cre *Dot1l*-cKO compared to ctrl at E12.5 (*n* = 6). (**C**) ChIP-qPCR analysis of H3K79me2 levels (% Input) in E14.5 ctrl (white bars) and *Foxg1*-Cre *Dot1l*-cKO (gray bars) forebrains, at the promotor of genes that are expressed in UL or DL cortical neurons (*n* = 4). (**D**) Upper panel: Representative image of immunostainings of cortical sections from E16.5 animals for PAX6, TBR2, TBR1, CTIP2 or SATB2 (green), RFP (red) and DAPI (blue) within their main expression domains, after *in utero* electroporation of DOT1L overexpression plasmid at E12.5. RFP marks electroporated cells, which assumingly overexpress DOT1L. Lower panel: Quantification of electroporated cells (RFP) that co-expressed PAX6, TBR2, TBR1, CTIP2 and SATB2, given as percentage of the total number of RFP/DOT1L OE cortical cells within the main expression domains/layers of the respective layer marker at E16.5 (PAX6 *n* = 3(ctrl)/4(*Dot1l*-cKO), TBR2 *n* = 3, TBR1 *n* = 3, CTIP2 *n* = 3, SATB2 *n* = 3). (**E**) Distribution of RFP-positive cells at E16.5 after *in utero* electroporation at E12.5 (left panel). The graph below the representative images shows the percentage of RFP-positive cells in each bin from the total RFP-positive cells determined in the 10 bins. Percentage of SATB2/RFP-positive cells (middle panel) or TBR1/RFP-positive cells (right panel) at E16.5 after *inutero* electroporation at E12.5. The graphs below the representative images show the percentage of SATB2/RFP- or TBR1/RFP-positive cells from the total RFP-positive cells in each bin (total RFP *n* = 5, RFP/SATB2 *n* = 3, RFP/TBR1 *n* = 6(ctrl)/5(*Dot1l-*cKO)). Data are presented as mean ± SEM. *P*-values were calculated using unpaired, two tailed Student's *t*-test: *P < 0.05, **P < 0.01, ***P < 0.001.

Phenotypically, *Dot1l-*cKO resulted in premature cell cycle exit of progenitors and altered numbers or distribution of neurons in the developing cortical plate. We identified moderate, but significant changes of the transcription levels of cell-cycle regulating genes *Ccnd1, E2f7, Vangl2*, and *Cenpj*, that might account for the observed differences of DOT1L-deficient progenitors. However, in addition to progenitor-assigned transcripts, our data revealed that subsets of genes usually expressed in differentiated neurons (for example *Satb2, Cux2, Sox 4, 11, 5*) were marked with H3K79me2 modification, and that their expression decreased upon loss of DOT1L (Figure 3E, 4A). This finding together with the moderate transcriptional changes of cell cycle regulating genes, fostered the hypothesis that DOT1L might activate specifically transcriptional programs of differentiated neurons. To test this hypothesis, we analyzed a set of genes, which are mainly expressed by differentiated neurons and that are described to mark either DL (*Tbr1*, Slit guidance ligand 1 (*Slit1*), Crystallin mu (*Crym*), Synaptotagmin 9 (*Syt9*), *Dkk3*, Synuclein alpha (*Scna*), Adenylate cyclase 1 (*Adcy1*)) or UL (*Satb2, Pou3f3* (POU domain class 3 transcription factor 3), *Cux1, Cux2*) (see Supplemental Table S3 for references) neurons. Interestingly, the transcripts expressed from UL neurons (*Satb2, Pou3f3, Cux1*, and *Cux2*) were marked with H3K79me2 already at E12.5 (Figure 6A), and their expression decreased upon *Dot1l*-cKO compared to controls. From the selected genes mainly expressed by DL neurons, only *Tbr1* was marked highly with H3K79me2, whereas the six other DL candidate genes were hardly marked. In support of our earlier hypothesis that strong enrichment of H3K79me2 is followed by transcriptional decrease upon *Dot1l-*cKO, expression of the selected UL genes significantly decreased already at E12.5, and also at E14.5 (Figure 6A, B). In contrast, upon *Dot1l* deletion, the set of DL markers either increased or did not change compared to controls at E14.5.

As protein expression of the UL marker genes is detectable mainly during late neurogenesis, but we observed transcription already at E12.5, we used qRTPCR to reveal the mRNA abundance of these candidates during cortical development. *In vivo* in the dorsal telencephalon, *Satb2, Pou3f3, Cux1*, and *Cux2* transcripts significantly increased from E11.5 to E13.5, and also toward E14.5-E15.5, but decreased in later embryonic and in the adult stage (Supplementary Figure S7A). As *Satb2, Pou3f3, Cux1*, and *Cux2* were marked with H3K79me2 already at E12.5 (Figure 6A), we hypothesized that transcription of these UL genes (1) might be marked epigenetically by H3K79me2 early in development and (2) would be transcriptionally influenced through DOT1L. H3K79me2 ChIP and qRTPCR of the region around the TSS of the selected UL and DL genes confirmed enrichment of H3K79me2 for the UL genes. This H3K79me2 enrichment was significantly lost in *Dot1l*-cKO compared to controls (Figure 6C). DL genes were hardly enriched for H3K79me2 modification (Figure 6A, C), and we could not detect significant alterations upon loss of DOT1L, except for *Tbr1*, which we described earlier as direct DOT1L target gene (5). This suggested that DOT1L and H3K79me2 directly affected gene expression of markers for differentiated neurons, especially of UL, but not DL marker gene expression.

As DOT1L influenced transcription of known genes conferring UL identity at E12.5, we overexpressed DOT1L at this developmental stage to analyze whether DOT1L function was instructive for neuronal differentiation. We used *in utero* electroporation (IUE) at E12.5 to overexpress DOT1L, harvested brains at E14.5 and E16.5, and visualized electroporated cells by expression of RFP, which was co-electroporated. We quantified the total number of RFP-positive cells and determined the percentage of RFP-positive cells that co-expressed either PAX6, TBR2, TBR1, CTIP2 or SATB2. Quantitation of the fraction of double positive cells was restricted to the main expression domains of the respective marker genes. We did not observe alterations in E14.5 brains that overexpress DOT1L compared to the empty vector control electroporation (Supplementary Figure S8A). In contrast, at E16.5, we observed decreased expression of TBR1 (Figure 6D) upon DOT1L-overexpression. In addition, we noticed that RFP-cells overexpressing DOT1L appeared in higher numbers in the lower half of the cortical plate at E16.5, and fewer numbers in the upper half of the cortex compared to control electroporated brains (Figure 6E). Moreover, a minor fraction of SATB2-positive cells populated deeper locations than in control brains (Figure 6E). We therefore quantified the numbers of TBR1- and SATB2-positive cells in the entire cortical plate, which we divided into 10 equal bins. SATB2-positive cells significantly increased in the proportion of SATB2/RFP-coexpressing cells in the deep cortical location upon DOT1L-overexpression. TBR1-positive cells decreased significantly in more superficial bins after DOT1L-overexpression as compared to controls (Figure 6E). As expected, these results showed that overexpression of DOT1L at E12.5 did not result in significant alterations of the number of progenitors expressing PAX6 and TBR2, but that increased DOT1L expression affected both, generation of TBR1- and SATB2-positive cells. The former is slightly inhibited through DOT1L, the latter facilitated. However, the overexpression of DOT1L most likely also impacted on distribution of TBR1 and SATB2 neurons, as the differences in numbers were comparably mild. Especially the SATB2-expression domain extended to the intermediate zone, indicating that the migration of SATB2-positive neurons might be hampered. In summary, the gain-of-function experiment suggested that DOT1L acted not exclusively by regulating cell cycle genes, but also by regulating specific developmental programs of neurons to ensure their proper generation as well as distribution in the cortical plate.

**Figure 7.**
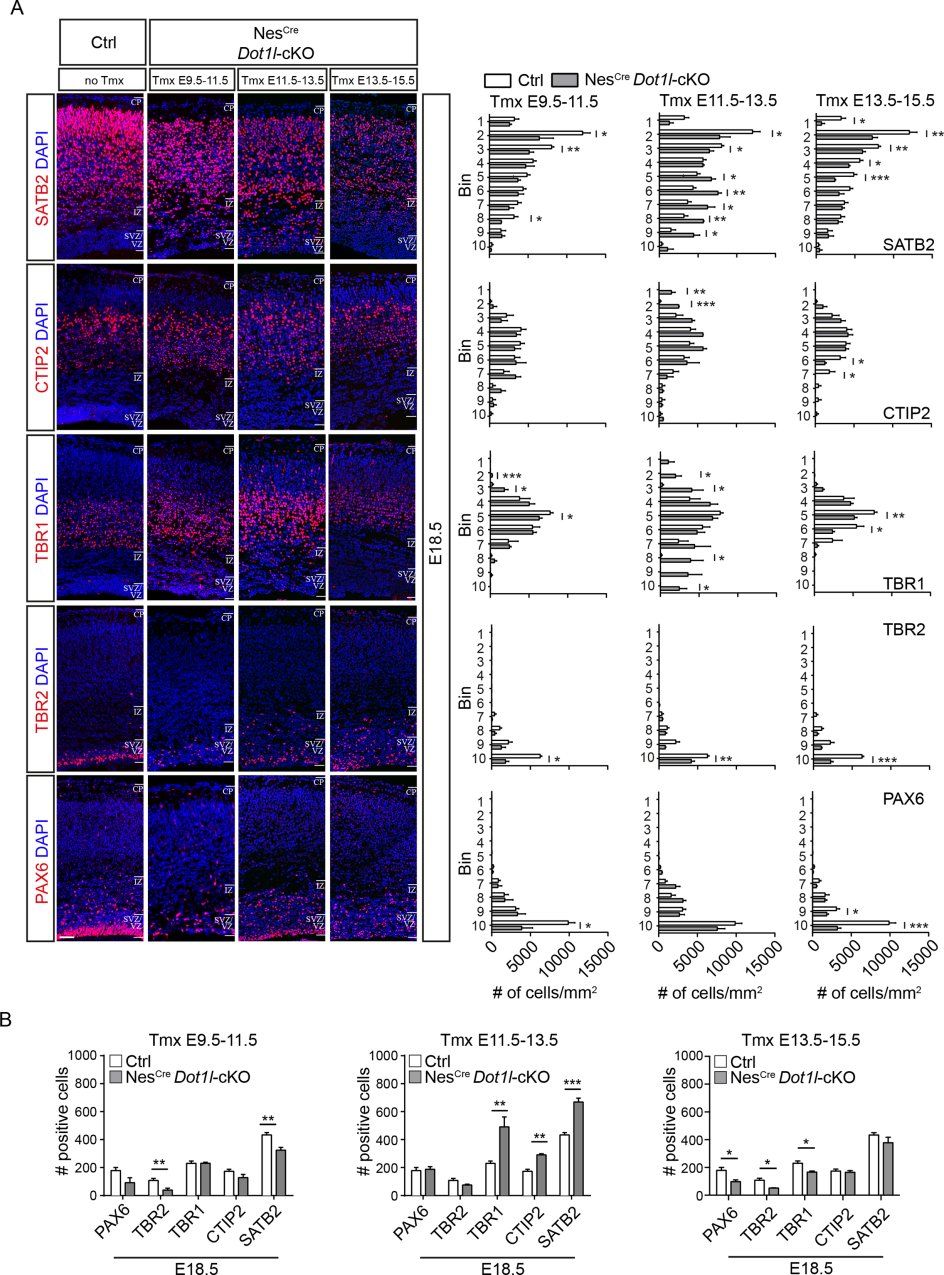
DOT1L primes SATB2-expression in the pre-neurogenic phase of cortical development. (**A**) Left panels: Representative immunostainings of E18.5 forebrains for PAX6, TBR2, TBR1, CITP2, and SATB2 (red), and DAPI (blue) of ctrl (no Tmx) and *Nes*-Cre *Dot1l*-cKO animals (three injections of Tmx at consecutive days starting at three different time points: Tmx E9.5-E11.5, Tmx E11.5–13.5, Tmx E13.5-E15.5). Scale bar: 50 μm. Right panels: Quantification of number of stained cells/mm^2^ within each individual bin out of 10 bins spanning the entire cortex in a width of 200 μm for ctrl and *Nes*-Cre*Dot1l*-cKO (ctrl *n* = 4 (white bars), Tmx E9.5-E11.5 *n* = 3, Tmx E11.5-E13.5 *n* = 3, Tmx E13.5-E15.5 *n* = 4 (gray bars)). (**B**) Total numbers of PAX6, TBR1, CTIP2 or SATB2-positive cells at E18.5 in 200 μm width of the cortex, as determined from the sum of positive cells for the respective markers counted in A, comparing ctrl (white bars) and *Nes*-Cre *Dot1l*-cKO with cKO induced at the indicated different time points (Tmx E9.5-E11.5, E11.5–13.5, E13.5-E15.5, gray bars).

The overexpression of DOT1L at E12.5 produced slightly, but significantly more SATB2 neurons with altered distribution in the cortical plate compared to controls. The *Satb2* gene had detectable H3K79me2 levels already at E12.5 (Figure 6A). We therefore hypothesized that an earlier DOT1L function might prime progenitors for a later SATB2 cell fate, implying that overexpression of DOT1L at E12.5 only affected a small, responsive progenitor fraction. We used an inducible *Nes*-Cre driver line to delete DOT1L before, around, and after E12.5 to resolve a potential critical time at which DOT1L affected generation of SATB2-positive neurons, or generally neurogenesis. *Dot1l-*cKO was induced by three consecutive tamoxifen injections at (i) E9.5, E10.5, and E11.5, (ii) E11.5, E12.5, and E13.5 and (iii) E13.5, E14.5, and E15.5 to interfere with DOT1L expression during three different phases of cortical neurogenesis (Supplementary Figure S8B). Brains were harvested at E18.5 and expression of PAX6, TBR2, TBR1, CTIP2, and SATB2 was quantified (Figure 7A, B). *Dot1l-*cKO before E12.5 reduced the numbers of PAX6- and TBR2-progenitors and let to differences in distribution of TBR1-neurons. CTIP2-neurons did not change, but as expected the numbers of SATB2-neurons significantly decreased. In contrast, induction of the *Dot1l-*cKO around E12.5 did not affect the numbers of progenitors, but led to a significant increased generation of TBR1-, CTIP2-, and SATB2-neurons, the latter of which also localized significantly more in DL. After E12.5, DOT1L-deficiency significantly reduced the numbers of progenitors and TBR1-neurons. SATB2-neurons did not change in numbers but were less frequent in UL positions.

In summary, data from all three *Dot1l*-cKO mouse lines and overexpression of DOT1L in the cerebral cortex presented in this study (Supplementary Figure S8C) support a model in which DOT1L has different functions during consecutive stages of cortical plate development. In essence, DOT1L maintained the progenitor pool, and it was necessary for proper distribution of DL and UL neurons. DOT1L primed a SATB2-neuronal cell fate through its activity before E12.5. One hallmark of this early DOT1L-dependent determination of SATB2 expression is the H3K79me2 enrichment in this gene in early progenitors.

## DISCUSSION

Our data show that the epigenetic chromatin modifier DOT1L is implicated in the development of the cortical plate, by maintaining the progenitor pool, and by affecting neuronal differentiation. DOT1L methylates histone H3 at K79, and if we correlate H3K79me2 pattern with DE genes, it is tempting to speculate that this epigenetic modification confers cellular commitment early during development, e.g. to prepare a SATB2 neuronal phenotype, which becomes apparent during later developmental stages.

The developmental advantage of using epigenetic traits to propagate information about potential cell fate is that it can be (i) established early in development, (ii) transmitted to future generations of progenitors, and (iii) kept at basal levels until full expression of a respective gene is needed. Thus, epigenetic modifications are important mechanisms to activate specific transcriptional programs in ESCs (51,52), and they also operate *in vivo* to shape the cerebral cortex. However, according to our RNA-seq data, H3K79me2 may not only have activating functions as suggested by various reports (17,53,54), but may also lead to transcriptional repression. We also reported reduced H3K79me2 levels at the *Tbr1* gene in mice deficient for the DOT1L-interaction partner AF9 (MLLT3, Super elongation complex subunit) (5). In this regard, H3K79me2 is distinct from activity-associated H3K4me3, which was reported to coincide with H3K79me2 (55–58). In our data only a subset of H3K4me3 marked genes carried also H3K79me2. We cannot rule out that H3K79me1 or me3 mark the rest of transcriptionally active genes. But our data most likely indicate that H3K79me2 is associated with a specific chromatin state, at genes with developmentally instructive functions during cortical development. H3K79me2 marks essential genes also in other developmentally restricted or committed stem cells (21,59,60). This co-occurrence can be interpreted that H3K79me2 has the potential to initiate, prime and/or maintain specific transcriptional programs that impact on cell fate decisions, in the cerebral cortex but also in other stem cells. However, we can only conclude that the histone modification correlates with the observed transcriptional changes, without ruling out that other DOT1L functions than methylation of H3K79 are involved and/or responsible.

Our results show that DOT1L functions at various stages of cortical development, and mainly activates transcriptional programs in progenitors and differentiated neurons. Others have reported that DOT1L influences the cell cycle and suggested functions in either G1/S- or M-phase. However, systematic mechanistic insight gained through transcriptome analyses and identification of *in vivo* target genes in mammalian cells are rare (61). We show here that DOT1L affects G1/S- and M-phase transitions in neural stem cells and increases expression of *Vangl2* and *Cenpj*. Both are novel DOT1L target genes that are associated with asymmetric cell division and cell fate decision in cortical development (49). VANGL2- and CENPJ-deficiency results in altered orientation of the cleavage plane, M-phase arrest, and premature differentiation. In addition, *Cenpj* mutations are associated with microcephaly (62). We observed an increased frequency of cleavage planes characteristic for asymmetric cell division in progenitors residing in the DOT1L-deficient VZ, which might indicate the relevance of the transcriptional regulation of *Vangl2* and *Cenpj* by DOT1L. It has been reported that H3K79me2 levels can vary during the cell cycle itself (61,63,64). However, these reported data are in part contradictory, and the cell cycle dynamics of H3K79me2 are not evolutionary conserved (65). It is thus impossible to ascertain whether variable H3K9me2 levels during different phases of the cell cycle are responsible for the phenotype of *Dot1l*-cKO forebrains.

Our data suggest to us that DOT1L might prime early born progenitors for adapting a specific phenotype for later developmental stages. This notion is supported by various observations: H3K79me2 was enriched in genes, which are transcribed in the progenitor zones and in UL (Figure 3). In addition, DOT1L deficiency reduced transcription of diverse *Sox* family member genes, in progenitors and differentiated neurons, and led to reduced levels of H3K79me2 at *Sox4, Sox11* and *Sox5* genes that are active in differentiated neurons (Figure 4). Similarly, loss of DOT1L reduced transcription and H3K79me2 levels of *Satb2, Pou3f3, Cux1*, and *Cux2*, all of which are mostly active in UL neurons, but these effects were not observed for selected DL genes (Figure 6). Furthermore, SATB2-expressing neurons significantly increased after overexpression of DOT1L at E12.5 *in vivo* (Figure 6) despite unchanged numbers of progenitors. Moreover, three mouse models in which DOT1L-deficiency occurred during pre-neurogenic phase before E12.5 had a defect in neurogenesis of SATB2-neurons, whereas TBR1 and CTIP2 neurons residing in DL distributed differently within the cortical plate compared to controls. This observation that interfering with DOT1L function in the pre-neurogenic phase leads to reduced numbers of SATB2-neurons is a strong argument in favor for an early priming of UL-cell fate through this epigenetic modifier (Supplementary Figure S8C).

Importantly, deletion of DOT1L in the three mouse models and at different developmental time points revealed that this enzyme has probably multiple functions during development. Compared to the loss of DOT1L in the pre-neurogenic phase (*Foxg1*-Cre, *Emx1*-Cre, *Nes*-Cre at E9.5–11.5), the cKO at later stages gave slightly different patterns in the genesis of progenitors and neurons. *Nes*-Cre induced cKO between E11.5 and E13.5 did not result in different numbers of progenitors. It is tempting to speculate that progenitors are permissive toward loss of DOT1L at this stage. However, as we also observed increasing cell numbers of TBR1-, CTIP2-, and SATB2-neurons it seems also likely that cells enter the cell cycle in higher numbers and that loss of DOT1L accounts for a facilitated exit for differentiation into DL and UL subtypes in these stages. This view is supported by the altered cell cycle in *Foxg1-*Cre cKO mice (Figure 2E–G), in which progenitors seem to follow this pattern. Increased differentiation into SATB2-expressing neurons around E12.5 might be favored as the early progenitors acquired the information to adopt this cell fate before the cKO was induced. Loss of DOT1L at the late neurogenic phase between E13.5 and E15.5 decreased the progenitor pool compared to controls. In contrast to the cKOs at earlier stages, we observed decreased generation of TBR1-expressing neurons during late neurogenesis upon DOT1L deletion. We speculate that this particular fraction of cells might localize to UL, according to our recently reported observation on AF9-deficient mice (5). AF9 interacts with DOT1L, and loss of AF9 increased TBR1-positive cells specifically in superficial positions. As progenitors received instructions to adopt a SATB2-cell fate during early development, the numbers of these neurons did not decrease upon *Dot1l*-cKO in this later developmental stage. However, as the distribution of SATB2-neurons significantly changed in the cortical plate upon DOT1L-deficiency in late neurogenesis, we conclude that DOT1L does not only function to balance the progenitor pool and to prime specific neuronal identity, but that it is also necessary for proper neuronal distribution in the developing cortex.

## DATA AVAILABILITY

Following deep sequencing data have been deposited in GEO: H3K79me2 E12.5: GSE95831, RNA-seq *Dot1l*-cKO vs ctrl: GSE95832, RNA-seq *Foxg1*-heterozygote vs ctrl: GSE95833. Following deep sequencing data have been deposited in NCBI BioProject: H3K79me2 E14.5: PRJNA282071, H3K4me3 E14.5: PRJNA287208.

## Supplementary Material

Supplementary Data

## References

[1] Qiao Y., Yang X., Jing N. Epigenetic regulation of early neural fate commitment. Cell Mol. Life Sci. 2016; 73:1399–1411. 26801220 10.1007/s00018-015-2125-6 PMC11108527

[2] Zahir F.R., Brown C.J. Epigenetic impacts on neurodevelopment: Pathophysiological mechanisms and genetic modes of action. Pediatr. Res. 2011; 69:92R–100R. 10.1203/PDR.0b013e318213565e 21293311

[3] Kubota T., Miyake K., Hariya N., Mochizuki K. Understanding the epigenetics of neurodevelopmental disorders and DOHaD. J. Dev. Orig. Health Dis. 2015; 6:96–104. 25708304 10.1017/S2040174415000057

[4] Britanova O., de Juan Romero C., Cheung A., Kwan K.Y., Schwark M., Gyorgy A., Vogel T., Akopov S., Mitkovski M., Agoston D. Satb2 is a postmitotic determinant for upper-layer neuron specification in the neocortex. Neuron. 2008; 57:378–392. 18255031 10.1016/j.neuron.2007.12.028

[5] Büttner N., Johnsen S.A., Kügler S., Vogel T. Af9/Mllt3 interferes with Tbr1 expression through epigenetic modification of histone H3K79 during development of the cerebral cortex. Proc. Natl. Acad. Sci. U.S.A. 2010; 107:7042–7047. 20348416 10.1073/pnas.0912041107 PMC2872432

[6] Martynoga B., Drechsel D., Guillemot F. Molecular control of neurogenesis: A view from the mammalian cerebral cortex. Cold Spring Harb. Perspect. Biol. 2012; 4:a008359. 23028117 10.1101/cshperspect.a008359 PMC3475166

[7] Shibata M., Gulden F.O., Sestan N. From trans to cis: transcriptional regulatory networks in neocortical development. Trends Genet. 2015; 31:77–87. 25624274 10.1016/j.tig.2014.12.004 PMC4382006

[8] Rakic P., Ayoub A.E., Breunig J.J., Dominguez M.H. Decision by division: making cortical maps. Trends Neurosci. 2009; 32:291–301. 19380167 10.1016/j.tins.2009.01.007 PMC3601545

[9] Hoch R.V., Rubenstein J.L.R., Pleasure S. Genes and signaling events that establish regional patterning of the mammalian forebrain. Semin. Cell Dev Biol. 2009; 20:378–386. 19560042 10.1016/j.semcdb.2009.02.005

[10] Chen C., Lee G.A., Pourmorady A., Sock E., Donoghue M.J. Orchestration of neuronal differentiation and progenitor pool expansion in the developing cortex by SoxC genes. J. Neurosci. 2015; 35:10629–10642. 26203155 10.1523/JNEUROSCI.1663-15.2015 PMC4510297

[11] Hutton S.R., Pevny L.H. SOX2 expression levels distinguish between neural progenitor populations of the developing dorsal telencephalon. Dev. Biol. 2011; 352:40–47. 21256837 10.1016/j.ydbio.2011.01.015

[12] Kwan K.Y., Lam M.M.S., Krsnik Ž., Kawasawa Y.I., Lefebvre V., Šestan N. SOX5 postmitotically regulates migration, postmigratory differentiation, and projections of subplate and deep-layer neocortical neurons. Proc. Natl. Acad. Sci. U.S.A. 2008; 105:16021–16026. 18840685 10.1073/pnas.0806791105 PMC2572944

[13] Dominguez M.H., Ayoub A.E., Rakic P. POU-III transcription factors (Brn1, Brn2, and Oct6) influence neurogenesis, molecular identity, and migratory destination of upper-layer cells of the cerebral cortex. Cereb. Cortex N. Y. 2013; 23:2632–2643. 10.1093/cercor/bhs252 PMC3792741 22892427

[14] Frantz G.D., McConnell S.K. Restriction of late cerebral cortical progenitors to an upper-layer fate. Neuron. 1996; 17:55–61. 8755478 10.1016/s0896-6273(00)80280-9

[15] Desai A.R., McConnell S.K. Progressive restriction in fate potential by neural progenitors during cerebral cortical development. Dev. Camb. Engl. 2000; 127:2863–2872. 10.1242/dev.127.13.2863 10851131

[16] Woo Park J., Kim K.-B., Kim J.-Y., Chae Y.-C., Jeong O.-S., Seo S.-B. RE-IIBP Methylates H3K79 and Induces MEIS1-mediated Apoptosis via H2BK120 Ubiquitination by RNF20. Sci. Rep. 2015; 5:12485. 26206755 10.1038/srep12485 PMC4513340

[17] Vlaming H., van Leeuwen F. The upstreams and downstreams of H3K79 methylation by DOT1L. Chromosoma. 2016; 125:593–605. 26728620 10.1007/s00412-015-0570-5

[18] Jones B., Su H., Bhat A., Lei H., Bajko J., Hevi S., Baltus G.A., Kadam S., Zhai H., Valdez R. The histone H3K79 methyltransferase Dot1L is essential for mammalian development and heterochromatin structure. PLoS Genet. 2008; 4:e1000190. 18787701 10.1371/journal.pgen.1000190 PMC2527135

[19] Feng Y., Yang Y., Ortega M.M., Copeland J.N., Zhang M., Jacob J.B., Fields T.A., Vivian J.L., Fields P.E. Early mammalian erythropoiesis requires the Dot1L methyltransferase. Blood. 2010; 116:4483–4491. 20798234 10.1182/blood-2010-03-276501 PMC3321834

[20] Nguyen A.T., Xiao B., Neppl R.L., Kallin E.M., Li J., Chen T., Wang D.-Z., Xiao X., Zhang Y. DOT1L regulates dystrophin expression and is critical for cardiac function. Genes Dev. 2011; 25:263–274. 21289070 10.1101/gad.2018511 PMC3034901

[21] Cattaneo P., Kunderfranco P., Greco C., Guffanti A., Stirparo G.G., Rusconi F., Rizzi R., Di Pasquale E., Locatelli S.L., Latronico M.V.G. DOT1L-mediated H3K79me2 modification critically regulates gene expression during cardiomyocyte differentiation. Cell Death Differ. 2016; 23:555–564. 25526092 10.1038/cdd.2014.199 PMC4986629

[22] Roidl D., Hellbach N., Bovio P.P., Villarreal A., Heidrich S., Nestel S., Grüning B.A., Boenisch U., Vogel T. DOT1L activity promotes proliferation and protects cortical neural stem cells from activation of ATF4-DDIT3-Mediated ER stress in vitro. Stem Cells Dayt Ohio. 2016; 34:233–245. 10.1002/stem.2187 26299268

[23] Hébert J.M., McConnell S.K. Targeting of cre to the Foxg1 (BF-1) locus mediates loxP recombination in the telencephalon and other developing head structures. Dev. Biol. 2000; 222:296–306. 10837119 10.1006/dbio.2000.9732

[24] Gorski J.A., Talley T., Qiu M., Puelles L., Rubenstein J.L.R., Jones K.R. Cortical excitatory neurons and glia, but not GABAergic neurons, are produced in the Emx1-expressing lineage. J. Neurosci. Off. J. Soc. Neurosci. 2002; 22:6309–6314. 10.1523/JNEUROSCI.22-15-06309.2002 PMC6758181 12151506

[25] Lagace D.C., Whitman M.C., Noonan M.A., Ables J.L., DeCarolis N.A., Arguello A.A., Donovan M.H., Fischer S.J., Farnbauch L.A., Beech R.D. Dynamic contribution of Nestin-Expressing stem cells to adult neurogenesis. J. Neurosci. 2007; 27:12623–12629. 18003841 10.1523/JNEUROSCI.3812-07.2007 PMC3718551

[26] Hellbach N., Weise S.C., Vezzali R., Wahane S.D., Heidrich S., Roidl D., Pruszak J., Esser J.S., Vogel T. Neural deletion of Tgfbr2 impairs angiogenesis through an altered secretome. Hum. Mol. Genet. 2014; 23:6177–6190. 24990151 10.1093/hmg/ddu338 PMC4222361

[27] Vezzali R., Weise S.C., Hellbach N., Machado V., Heidrich S., Vogel T. The FOXG1/FOXO/SMAD network balances proliferation and differentiation of cortical progenitors and activates Kcnh3 expression in mature neurons. Oncotarget. 2016; 7:37436–37455. 27224923 10.18632/oncotarget.9545 PMC5122323

[28] Artegiani B., Lange C., Calegari F. Expansion of embryonic and adult neural stem cells by in utero electroporation or viral stereotaxic injection. J. Vis. Exp. JoVE. 2012; 68:e4093. 10.3791/4093 PMC3490322 23070124

[29] Afgan E., Baker D., van den Beek M., Blankenberg D., Bouvier D., Čech M., Chilton J., Clements D., Coraor N., Eberhard C. The Galaxy platform for accessible, reproducible and collaborative biomedical analyses: 2016 update. Nucleic Acids Res. 2016; 44:W3–W10. 27137889 10.1093/nar/gkw343 PMC4987906

[30] Krueger F. Trim Galore Babraham Bioinformatics. http://www.bioinformatics.babraham.ac.uk/projects/trim_galore/.

[31] Kim D., Pertea G., Trapnell C., Pimentel H., Kelley R., Salzberg S.L. TopHat2: accurate alignment of transcriptomes in the presence of insertions, deletions and gene fusions. Genome Biol. 2013; 14:R36. 23618408 10.1186/gb-2013-14-4-r36 PMC4053844

[32] Yates A., Akanni W., Amode M.R., Barrell D., Billis K., Carvalho-Silva D., Cummins C., Clapham P., Fitzgerald S., Gil L. Ensembl 2016. Nucleic Acids Res. 2016; 44:D710–D716. 26687719 10.1093/nar/gkv1157 PMC4702834

[33] Anders S., Pyl P.T., Huber W. HTSeq—a Python framework to work with high-throughput sequencing data. Bioinformatics. 2015; 31:166–169. 25260700 10.1093/bioinformatics/btu638 PMC4287950

[34] Love M.I., Huber W., Anders S. Moderated estimation of fold change and dispersion for RNA-seq data with DESeq2. Genome Biol. 2014; 15:550. 25516281 10.1186/s13059-014-0550-8 PMC4302049

[35] Langmead B., Salzberg S.L. Fast gapped-read alignment with Bowtie 2. Nat. Methods. 2012; 9:357–359. 22388286 10.1038/nmeth.1923 PMC3322381

[36] Zhang Y., Liu T., Meyer C.A., Eeckhoute J., Johnson D.S., Bernstein B.E., Nusbaum C., Myers R.M., Brown M., Li W. Model-based Analysis of ChIP-Seq (MACS). Genome Biol. 2008; 9:R137. 18798982 10.1186/gb-2008-9-9-r137 PMC2592715

[37] Ross-Innes C.S., Stark R., Teschendorff A.E., Holmes K.A., Ali H.R., Dunning M.J., Brown G.D., Gojis O., Ellis I.O., Green A.R. Differential oestrogen receptor binding is associated with clinical outcome in breast cancer. Nature. 2012; 481:389–393. 22217937 10.1038/nature10730 PMC3272464

[38] Ramírez F., Ryan D.P., Grüning B., Bhardwaj V., Kilpert F., Richter A.S., Heyne S., Dündar F., Manke T. deepTools2: a next generation web server for deep-sequencing data analysis. Nucleic Acids Res. 2016; 44:W160–W165. 27079975 10.1093/nar/gkw257 PMC4987876

[39] Eagleson K.L., Schlueter McFadyen-Ketchum L.J., Ahrens E.T., Mills P.H., Does M.D., Nickols J., Levitt P. Disruption of Foxg1 expression by knock-in of cre recombinase: effects on the development of the mouse telencephalon. Neuroscience. 2007; 148:385–399. 17640820 10.1016/j.neuroscience.2007.06.012 PMC2194757

[40] Kawaguchi D., Sahara S., Zembrzycki A., O’Leary D.D.M. Generation and analysis of an improved Foxg1-IRES-Cre driver mouse line. Dev. Biol. 2016; 412:139–147. 26896590 10.1016/j.ydbio.2016.02.011 PMC5895454

[41] Molyneaux B.J., Arlotta P., Menezes J.R.L., Macklis J.D. Neuronal subtype specification in the cerebral cortex. Nat. Rev. Neurosci. 2007; 8:427–437. 17514196 10.1038/nrn2151

[42] Sessa A., Mao C., Hadjantonakis A.-K., Klein W.H., Broccoli V. Tbr2 directs conversion of radial glia into basal precursors and guides neuronal amplification by indirect neurogenesis in the developing neocortex. Neuron. 2008; 60:56–69. 18940588 10.1016/j.neuron.2008.09.028 PMC2887762

[43] Sarnat H.B., Flores-Sarnat L. Role of Cajal-Retzius and subplate neurons in cerebral cortical development. Semin. Pediatr. Neurol. 2002; 9:302–308. 12523554 10.1053/spen.2002.32506

[44] Bernstein B.E., Kamal M., Lindblad-Toh K., Bekiranov S., Bailey D.K., Huebert D.J., McMahon S., Karlsson E.K., Kulbokas E.J., Gingeras T.R. Genomic maps and comparative analysis of histone modifications in human and mouse. Cell. 2005; 120:169–181. 15680324 10.1016/j.cell.2005.01.001

[45] Zhou Q., Anderson D.J. The bHLH transcription factors OLIG2 and OLIG1 couple neuronal and glial subtype specification. Cell. 2002; 109:61–73. 11955447 10.1016/s0092-8674(02)00677-3

[46] Alao J.P., Gamble S.C., Stavropoulou A.V., Pomeranz K.M., Lam E.W.-F., Coombes R.C., Vigushin D.M. The cyclin D1 proto-oncogene is sequestered in the cytoplasm of mammalian cancer cell lines. Mol. Cancer. 2006; 5:7. 16503970 10.1186/1476-4598-5-7 PMC1388232

[47] Tadeu A.M.B., Ribeiro S., Johnston J., Goldberg I., Gerloff D., Earnshaw W.C. CENP-V is required for centromere organization, chromosome alignment and cytokinesis. EMBO J. 2008; 27:2510–2522. 18772885 10.1038/emboj.2008.175 PMC2532784

[48] Delaunay D., Cortay V., Patti D., Knoblauch K., Dehay C. Mitotic spindle asymmetry: a Wnt/PCP-regulated mechanism generating asymmetrical division in cortical precursors. Cell Rep. 2014; 6:400–414. 24412369 10.1016/j.celrep.2013.12.026

[49] Garcez P.P., Diaz-Alonso J., Crespo-Enriquez I., Castro D., Bell D., Guillemot F. Cenpj/CPAP regulates progenitor divisions and neuronal migration in the cerebral cortex downstream of Ascl1. Nat. Commun. 2015; 6:6474. 25753651 10.1038/ncomms7474 PMC4366522

[50] Bibel M., Richter J., Schrenk K., Tucker K.L., Staiger V., Korte M., Goetz M., Barde Y.-A. Differentiation of mouse embryonic stem cells into a defined neuronal lineage. Nat. Neurosci. 2004; 7:1003–1009. 15332090 10.1038/nn1301

[51] Hirabayashi Y., Gotoh Y. Epigenetic control of neural precursor cell fate during development. Nat. Rev. Neurosci. 2010; 11:377–388. 20485363 10.1038/nrn2810

[52] Albert M., Kalebic N., Florio M., Lakshmanaperumal N., Haffner C., Brandl H., Henry I., Huttner W.B. Epigenome profiling and editing of neocortical progenitor cells during development. EMBO J. 2017; 36:2642–2658. 28765163 10.15252/embj.201796764 PMC5579386

[53] Zhang W., Xia X., Jalal D.I., Kuncewicz T., Xu W., Lesage G.D., Kone B.C. Aldosterone-sensitive repression of ENaCalpha transcription by a histone H3 lysine-79 methyltransferase. Am. J. Physiol. Cell Physiol. 2006; 290:C936–C946. 16236820 10.1152/ajpcell.00431.2005 PMC3009459

[54] Reisenauer M.R., Wang S.W., Xia Y., Zhang W. Dot1a contains three nuclear localization signals and regulates the epithelial Na+ channel (ENaC) at multiple levels. Am. J. Physiol. Renal Physiol. 2010; 299:F63–F76. 20427473 10.1152/ajprenal.00105.2010 PMC2904171

[55] Lee J.-S., Shukla A., Schneider J., Swanson S.K., Washburn M.P., Florens L., Bhaumik S.R., Shilatifard A. Histone crosstalk between H2B monoubiquitination and H3 methylation mediated by COMPASS. Cell. 2007; 131:1084–1096. 18083099 10.1016/j.cell.2007.09.046

[56] Barski A., Cuddapah S., Cui K., Roh T.-Y., Schones D.E., Wang Z., Wei G., Chepelev I., Zhao K. High-resolution profiling of histone methylations in the human genome. Cell. 2007; 129:823–837. 17512414 10.1016/j.cell.2007.05.009

[57] Ernst J., Kheradpour P., Mikkelsen T.S., Shoresh N., Ward L.D., Epstein C.B., Zhang X., Wang L., Issner R., Coyne M. Mapping and analysis of chromatin state dynamics in nine human cell types. Nature. 2011; 473:43–49. 21441907 10.1038/nature09906 PMC3088773

[58] Black J.C., Van Rechem C., Whetstine J.R. Histone lysine methylation dynamics: establishment, regulation, and biological impact. Mol. Cell. 2012; 48:491–507. 23200123 10.1016/j.molcel.2012.11.006 PMC3861058

[59] Onder T.T., Kara N., Cherry A., Sinha A.U., Zhu N., Bernt K.M., Cahan P., Marcarci B.O., Unternaehrer J., Gupta P.B. Chromatin-modifying enzymes as modulators of reprogramming. Nature. 2012; 483:598–602. 22388813 10.1038/nature10953 PMC3501145

[60] Jackson S.A., Olufs Z.P.G., Tran K.A., Zaidan N.Z., Sridharan R. Alternative routes to induced pluripotent stem cells revealed by reprogramming of the neural lineage. Stem Cell Rep. 2016; 6:302–311. 10.1016/j.stemcr.2016.01.009 PMC4788781 26905202

[61] Kim W., Kim R., Park G., Park J.-W., Kim J.-E. Deficiency of H3K79 histone methyltransferase Dot1-like protein (DOT1L) inhibits cell proliferation. J. Biol. Chem. 2012; 287:5588–5599. 22190683 10.1074/jbc.M111.328138 PMC3285333

[62] Bond J., Roberts E., Springell K., Lizarraga S.B., Lizarraga S., Scott S., Higgins J., Hampshire D.J., Morrison E.E., Leal G.F. A centrosomal mechanism involving CDK5RAP2 and CENPJ controls brain size. Nat. Genet. 2005; 37:353–355. 15793586 10.1038/ng1539

[63] Ooga M., Inoue A., Kageyama S., Akiyama T., Nagata M., Aoki F. Changes in H3K79 methylation during preimplantation development in mice. Biol. Reprod. 2008; 78:413–424. 18003948 10.1095/biolreprod.107.063453

[64] Schulze J.M., Jackson J., Nakanishi S., Gardner J.M., Hentrich T., Haug J., Johnston M., Jaspersen S.L., Kobor M.S., Shilatifard A. Linking cell cycle to histone modifications: SBF and H2B monoubiquitination machinery and cell-cycle regulation of H3K79 dimethylation. Mol. Cell. 2009; 35:626–641. 19682934 10.1016/j.molcel.2009.07.017 PMC3222332

[65] Kim W., Choi M., Kim J.-E. The histone methyltransferase Dot1/DOT1L as a critical regulator of the cell cycle. Cell Cycle. 2014; 13:726–738. 24526115 10.4161/cc.28104 PMC3979909

