# Supplementary material for: DOT1L promotes progenitor proliferation and primes neuronal layer identity in the developing cerebral cortex
Source: Nucleic Acids Res. 2018 Oct 17;47(1):168–83. doi: 10.1093/nar/gky953 (PMC6326801; doi:10.1093/nar/gky953)
Supplement: Supplementary Data [file gky953_supplemental_files.pdf]

A

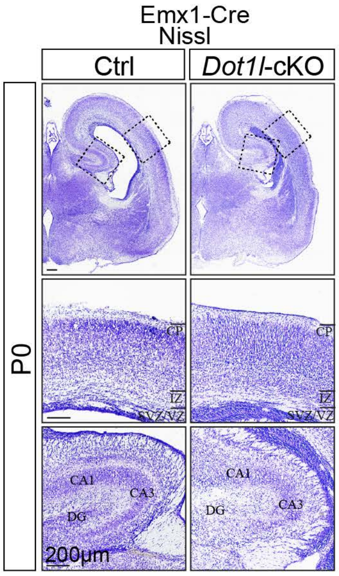

Supplemental_Fig_S2

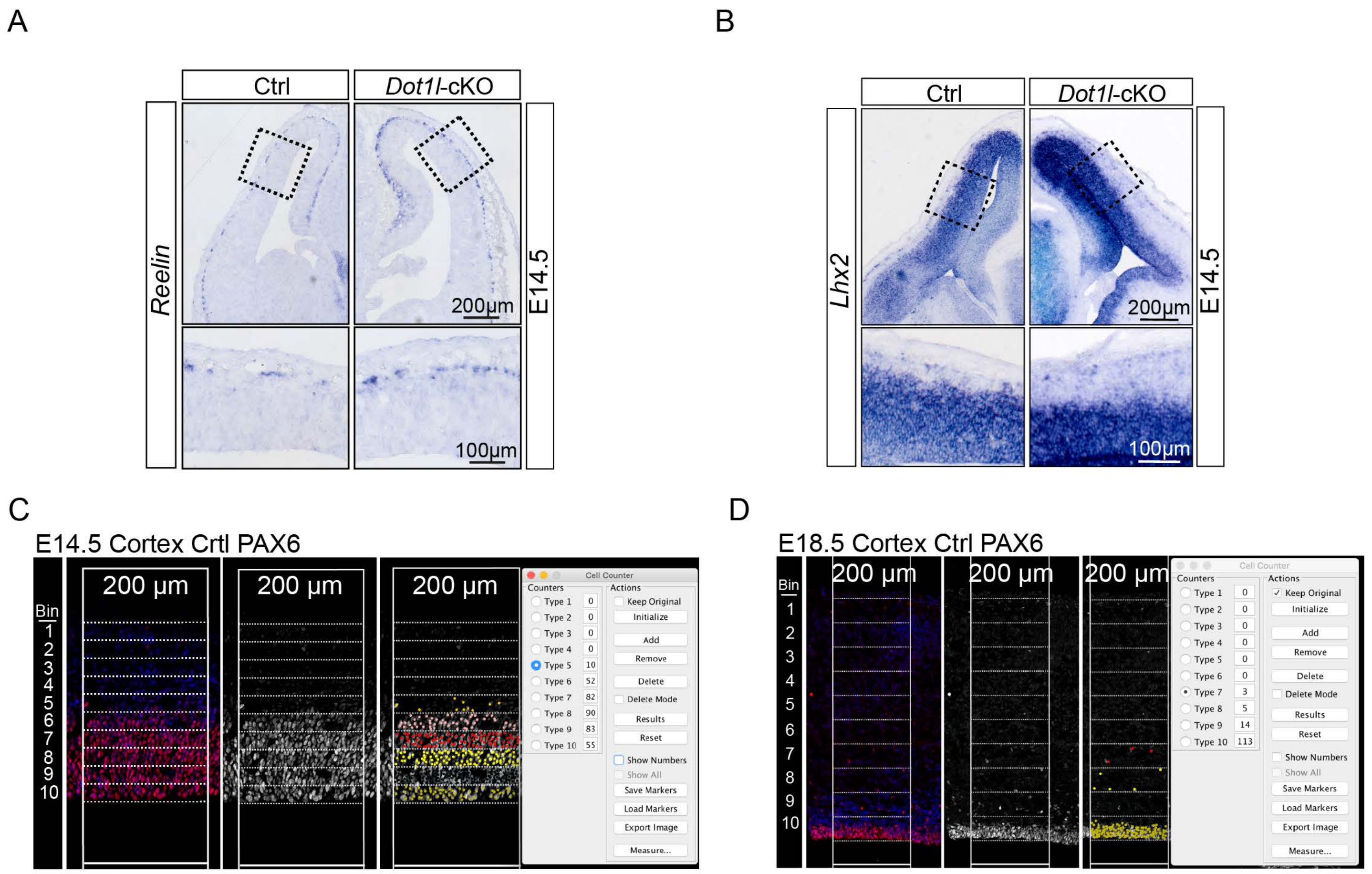

Supplemental_Fig_S3

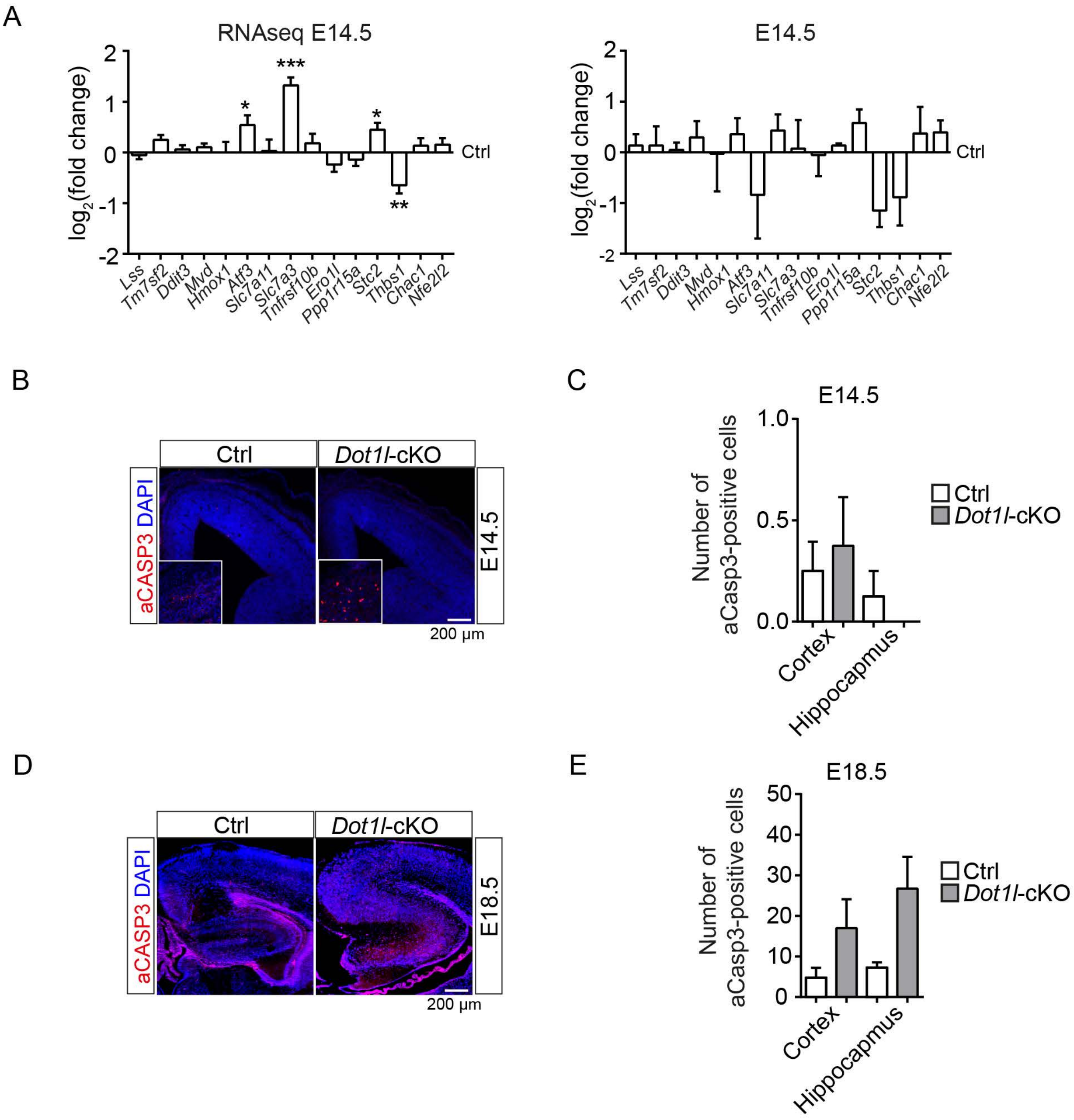

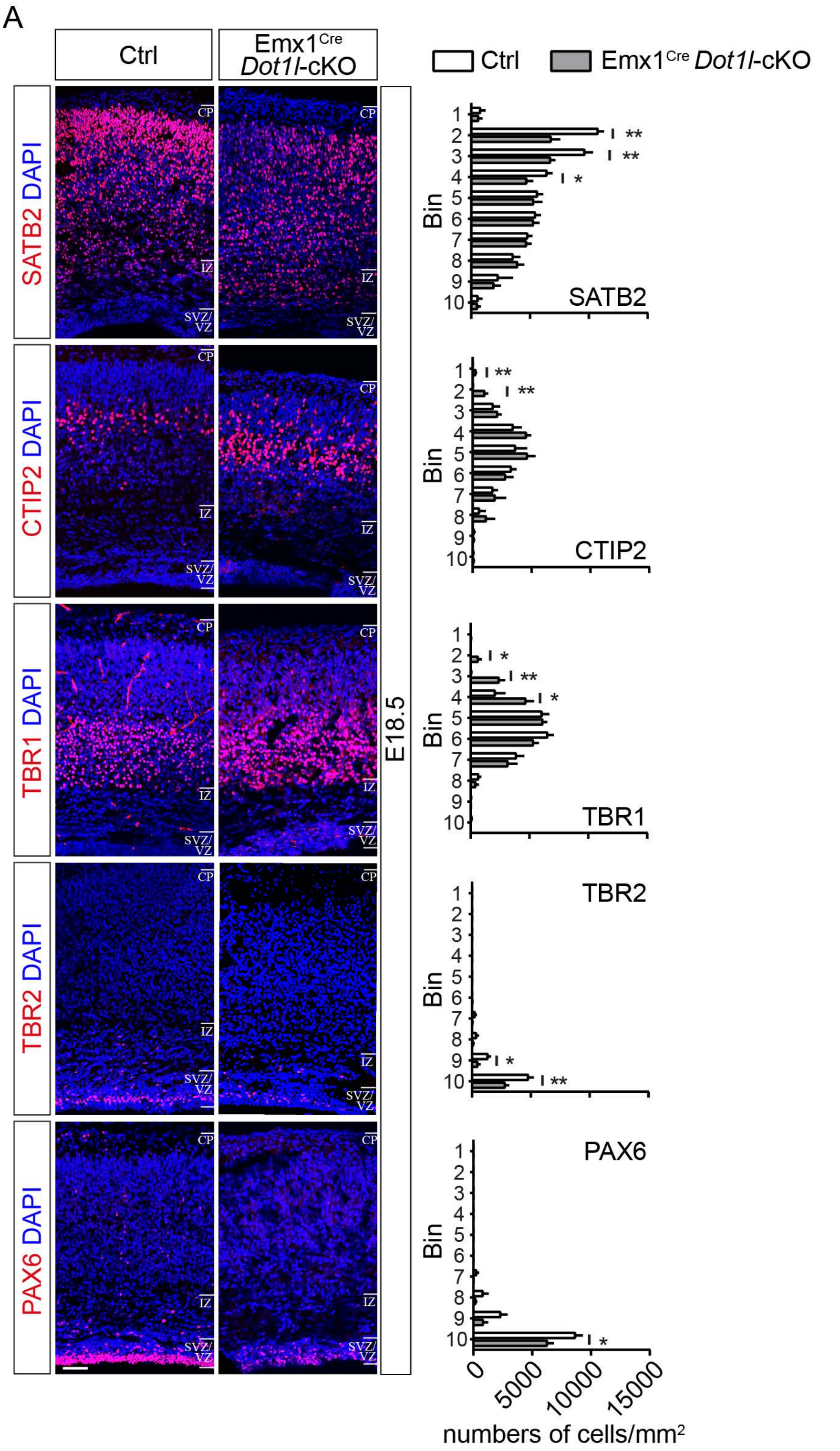
A
Ctrl
Emx1Cre Dot1l-cKO
SATB2 DAPI
CTIP2 DAPI
TBR1 DAPI
TBR2 DAPI
PAX6 DAPI
CP
IZ
SVZ/VZ
E18.5
Ctrl
Emx1Cre Dot1l-cKO
Bin
SATB2
CTIP2
TBR1
TBR2
PAX6
numbers of cells/mm2
0
5000
10000
15000
B
Ctrl
EmxCre Dot1l-cKO
positive cells
PAX6
TBR2
TBR1
CTIP2
SATB2
E18.5

Supplemental_Fig_S5

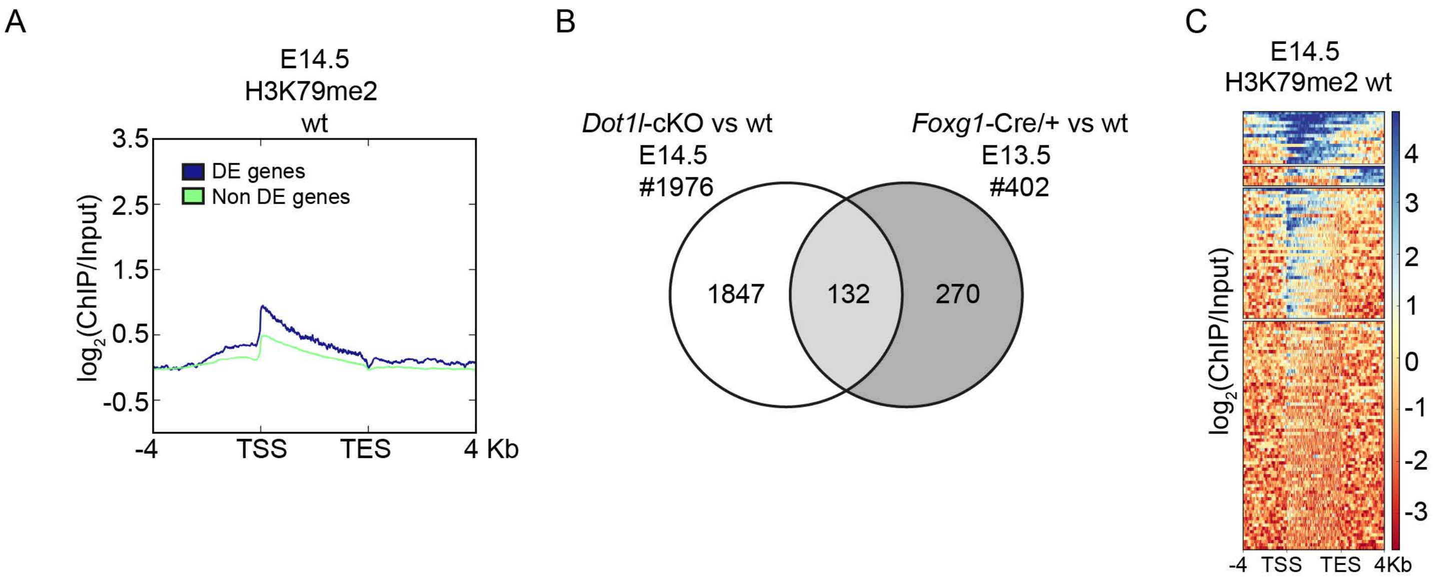

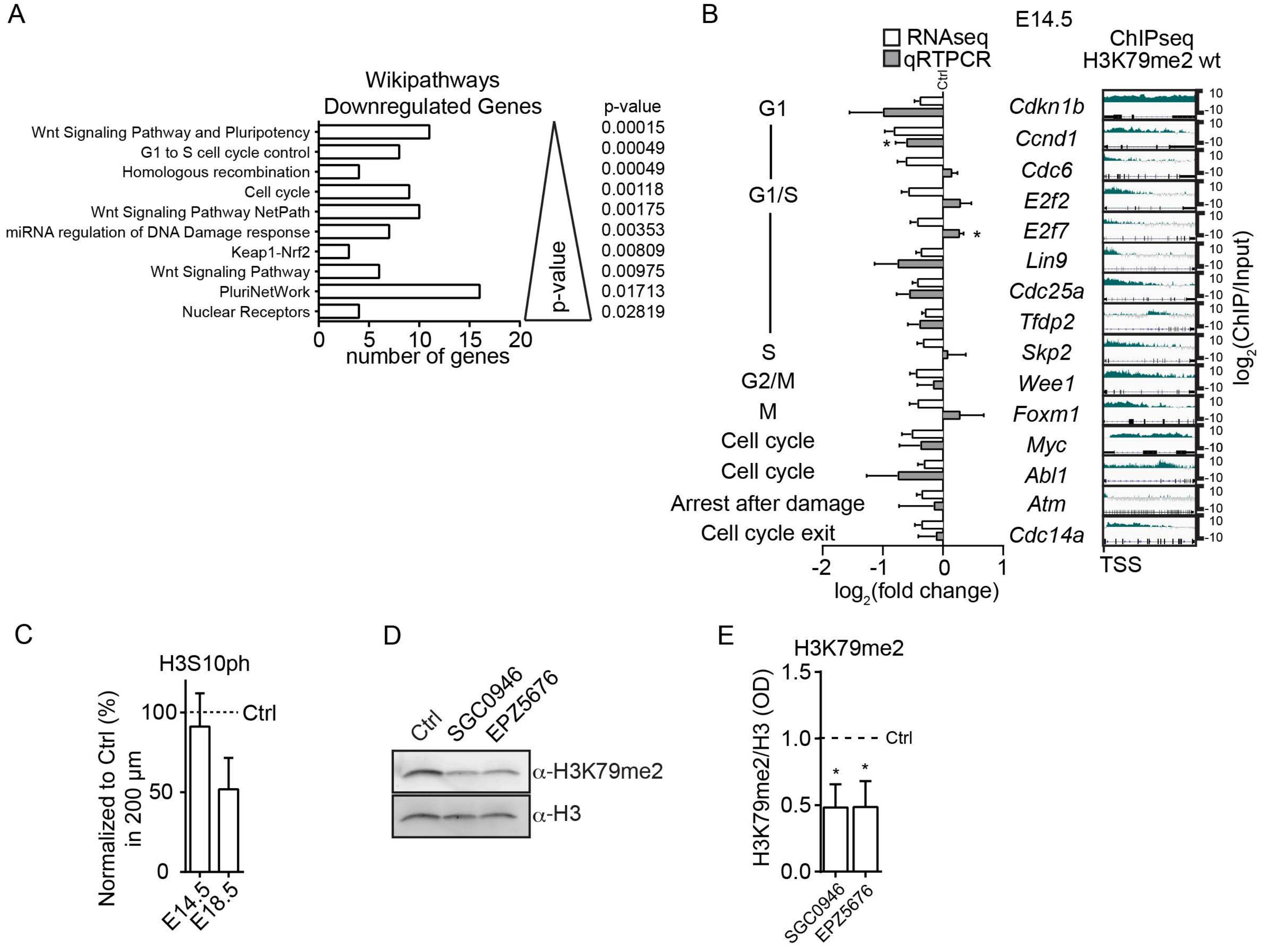
A
Wikipathways
Downregulated Genes
p-value
Wnt Signaling Pathway and Pluripotency 0.00015
G1 to S cell cycle control 0.00049
Homologous recombination 0.00049
Cell cycle 0.00118
Wnt Signaling Pathway NetPath 0.00175
miRNA regulation of DNA Damage response 0.00353
Keap1-Nrf2 0.00809
Wnt Signaling Pathway 0.00975
PluriNetWork 0.01713
Nuclear Receptors 0.02819
p-value
0 5 10 15 20
number of genes
B
E14.5
RNAseq
qRTPCR
Ctrl
ChIPseq
H3K79me2 wt
G1
G1/S
S
G2/M
M
Cell cycle
Cell cycle
Arrest after damage
Cell cycle exit
Cdkn1b
Ccnd1
Cdc6
E2f2
E2f7
Lin9
Cdc25a
Tfdp2
Skp2
Wee1
Foxm1
Myc
Abl1
Atm
Cdc14a
-2 -1 0 1
log2(fold change)
TSS
log2(ChIP/Input)
C
H3S10ph
Normalized to Ctrl (%) in 200 µm
Ctrl
E14.5
E18.5
D
Ctrl
SGC0946
EPZ5676
α-H3K79me2
α-H3
E
H3K79me2
H3K79me2/H3 (OD)
Ctrl
SGC0946
EPZ5676

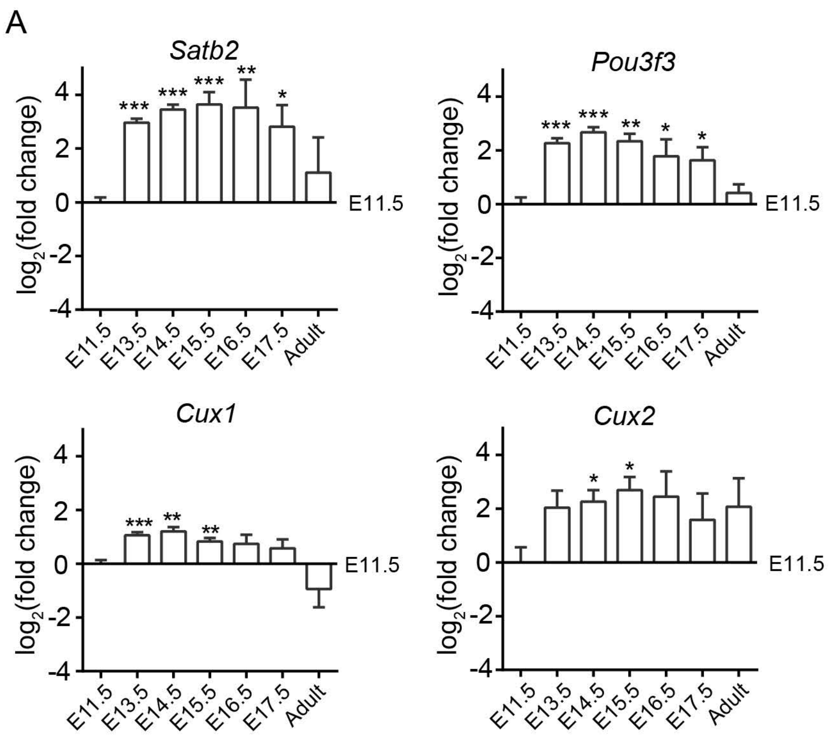
A
Satb2
Pou3f3
Cux1
Cux2
log2(fold change)
4
2
0
-2
-4
E11.5
E11.5
E13.5
E14.5
E15.5
E16.5
E17.5
Adult
***
**
*

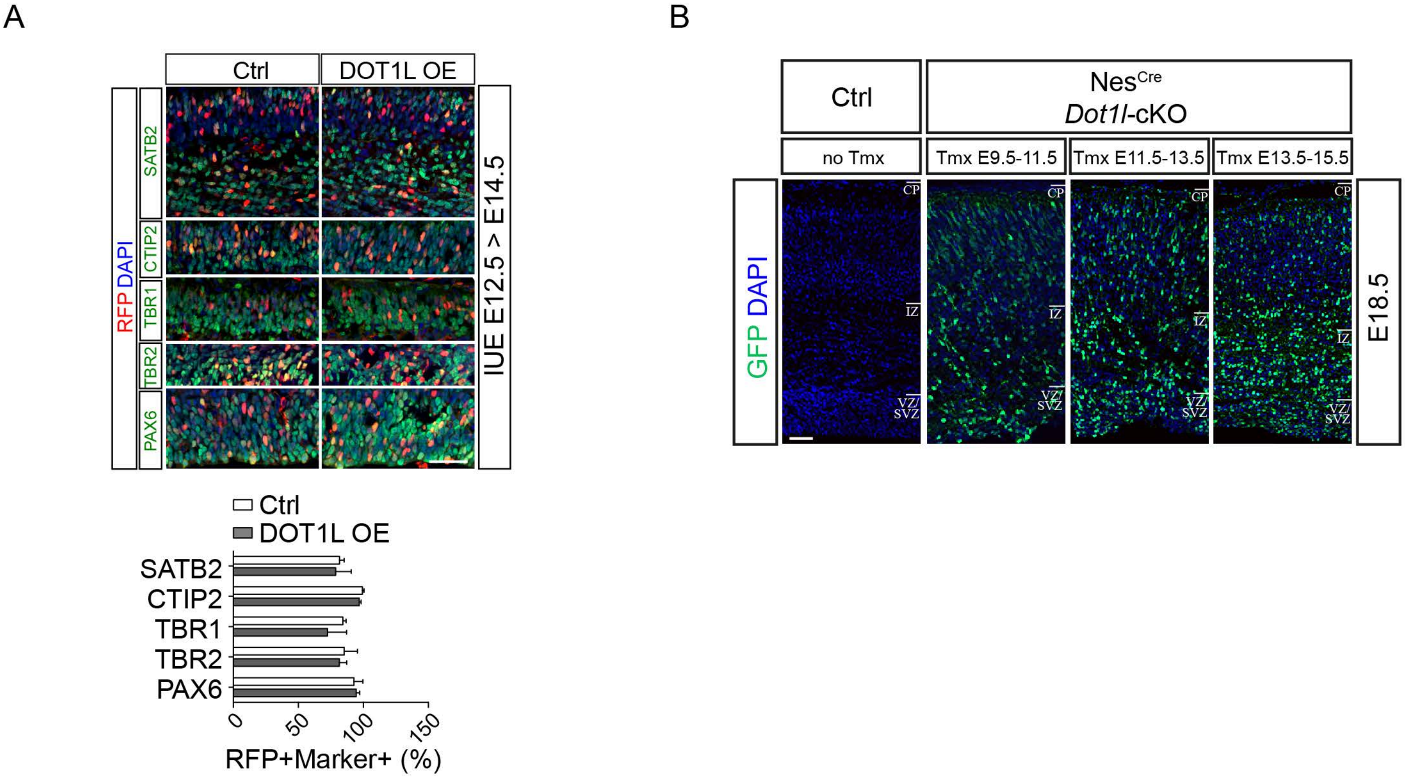

C

E18.5

	FoxG1^{Cre} *Dot1l*-cKO	Emx^{Cre} *Dot1l*-cKO	Nes^{Cre} *Dot1l*-cKO E9.5-E11.5	Nes^{Cre} *Dot1l*-cKO E11.5-E13.5	Nes^{Cre} *Dot1l*-cKO E13.5-E15.5
PAX6	genesis down	genesis	genesis	n.c.	genesis down
TBR2	n.a.	genesis down	genesis down	genesis	genesis down
TBR1	distribution	distribution	distribution	distribution genesis up	genesis down
CTIP2	distribution	distribution	n.c.	distribution genesis up	distribution
SATB2	genesis down	genesis down	genesis down	distribution genesis up	distribution

Supplemental Table S1

Target	Purpose	forward	reverse
Abl1	qRTPCR	CTGGAAAAGTTCTTGGGGACCA	GGCTCAAAGTCAGATGCCACT
Adcy1	qRTPCR	CCCAGCCTAAGACGGATCAC	GGGTCACGTCATTGGACCAT
Ankrd6	qRTPCR	CTGAGACTGGACGGAAGGTT	CCGTGGGAAAGTCCATAGGC
Arx	qRTPCR	TCCAACCCTCCAGGAGAGAG	CAGCTCAGCCTCGAACGG
Atf3	qRTPCR	CCCCTGGAGATGTCAGTCAC	TTCTGCAGGCACTCTGTCTT
Atm	qRTPCR	AGGAACCAGTTACCATGAATCG	TCTTCAACTTCTTTCACCCTGA
Ccnd1	qRTPCR	GAGATTGTGCCATCCATGCG	GGAAGACCTCCTCTTCGCAC
Cdc14a	qRTPCR	TACACCTCTTTCGACCAGCG	GTCAAGGACGGTGAGGTTGT
Cdc25a	qRTPCR	CAAACCTTGCCGATCGTTGC	AGGGATAGAGACTGGGATGGA
Cdc6	qRTPCR	CACTCCGTGTGTGGACGTAA	AACAGGGAGATAACCGGGGA
Cdkn1b	qRTPCR	TTCGACGCCAGACGTAAACA	GGCAGATGGTTTAAGAGTGCCT
Cenpj	qRTPCR	GTGACAGTGTTCGATCTCAGGT	ATTCGAGCCAGCTCTCTTGC
Cenpv	qRTPCR	AGCTGCTGCTGGACACCTTT	ACCCTTCAGGAGTTTGAACCG
Chac1	qRTPCR	CTATAGCCGACGGTTCTGGC	TTGGTATGCCACACCCCAAG
Crym	qRTPCR	TGCAAGGAGATGTTCGGGTC	CATCCAGTTCTCGCCAGTCA
Cux1	qRTPCR	GCCTCTCACTCCGTCTCAAT	TGTTCGCTCTCATCTTGCCT
Cux2	qRTPCR	CGGACATCCATCTCTTAGCCC	CTGAACACCGCCGTACTCC
Cyp39a1	qRTPCR	GGCTTTGAGTATGGCTCCCA	CAGCCCAAAGTACGACCAGT
Ddit3	qRTPCR	CAGAGCCAGAATAACAGCCG	TGGACCAGGTTCTGCTTTCA
Dkk3	qRTPCR	CACAATGAGACCAGCACGGA	CACCTGTCCACTCTGGTTGT
Dkk3	qRTPCR	CACAATGAGACCAGCACGGA	CACCTGTCCACTCTGGTTGT
Dot1l	qRTPCR	TGAGGCTCAAGTCGCCTGT	GCATCATGGTGCTTGTCGTA
E2f2	qRTPCR	AAATTGTGCGATGTGCACCC	CGGAATTCAGGGACCGTAGG
E2f7	qRTPCR	GCAGCGACAATGGAGGTGAA	GGCTCGTTCTTCATCGGTGT
Ero1l	qRTPCR	TCTCTGAGTGAGGAGACCCA	GTAGCGCTCAGGGTTAAGGA
Foxm1	qRTPCR	CAGACGCGGCCTGTGAG	TGGTTCTCATCGTGAGGCTG
Fzd2	qRTPCR	CCTCAAGGTGCCGTCCTATC	CAACACCGACCATGTGAGGA
Fzd3	qRTPCR	CGGCATCTGGGAGACAACAT	CTGCCGGCTCTCATTCACTA
Glra2	qRTPCR	TGTACCGGCAGCTAGTGAAC	AGGAGATAGGGTCTGCGAGG
Hmox1	qRTPCR	AGAACCAGCCTGAACTAGCC	CAGCATTCTCGGCTTGGATG
Igf2bp1	qRTPCR	CGTCACCTACTCTAACCGGG	AGGACCTTGCGTTATCTGCT
Lhx2	qRTPCR	CGCTGGGTCTTCCCTACTAC	ATCCAGGTGTTCAGCATCGT
Lhx5	qRTPCR	TGAGGGTCATTCAGGTGTGG	ATCTCAGACTCGTCCAAGCG
Lin9	qRTPCR	TGACGAGAGCTCTTCAGCAAA	TGCCTTTCCAAACAGGAGTTTTC
Lss	qRTPCR	AGGGCACGTGTCTGCG	GTCCTGCAGGTAGGTCCAC
Mvd	qRTPCR	GTAATCGGTGCTGGGCTCT	TTGCTGATGACGGCTGTTGT
Myc	qRTPCR	CGCGATCAGCTCTCCTGAAA	CCAGATATCCTCACTGGGCG
Myc	qRTPCR	CGCGATCAGCTCTCCTGAAA	CCAGATATCCTCACTGGGCG
Nfe2l2	qRTPCR	GCCCTCAGCATGATGGACTT	TCTATGTCTTGCCTCCAAAGGA
Nfib	qRTPCR	CCCGTCCTGATTGATCGCAT	AATTGCACGGACGTGAGGAA
Nlk	qRTPCR	GGGGTCCTCATAAACAGCCA	GGCTCAAAGTCACTGGTGTAGA
Otx1	qRTPCR	TAGATGGTGAAAAGCCGCGA	ACATCATGCTAACAGCCGGG
Pou3f3	qRTPCR	GAAGGGTAGGTACGTCCAGC	GGCGGAATCTTTAGGCATGG
Ppp1r15a	qRTPCR	CCTCTAAAAGCTCGGAAGGTACA	GATCTCGTGCAAACTGCTCC
Ppp1r1b	qRTPCR	CTCCGCGCAGCACGC	GAAAAGCATGGCAGGGGTTG
Prkcq	qRTPCR	ACTTTGACTGTGGGACCTGC	GGTGGGTACATGGTTGGCTT
Reln	qRTPCR	TGGCAACCCATCCTTCCACCTCT	CACGAGCTGCCAGGAATCGGAC
Satb2	qRTPCR	TGACCCAGGACCTTAAACGC	TGGAGACAAAGTTCCCACCG
Scna	qRTPCR	CTGCCCTTGCCTCTTTCATTG	CTCCCTCCTTGGCCTTTGAA
Skp2	qRTPCR	CTCCAACACCTCTCGCTCAG	ATGGTGGTGAAATAGGCGCA
Slc7a11	qRTPCR	TTTACCACCATCAGTGCGGA	GAACACACCACCGTTCATGG
Slc7a3	qRTPCR	GGCGCATCTCCCAATTTCAG	CGATCCACCTGAGCCTTCTT
Slit1	qRTPCR	TGCCCATTTTCCTCCTCACA	TACCTAGATGCTCCCAGCGA
Sox1	qRTPCR	GGGCTACGGCGGCATC	AGGGCCCCCGACGAC
Sox10	qRTPCR	AGATCCAGTTCCGTGTCAATAA	GCGAGAAGAAGGCTAGGTG
Sox11	qRTPCR	CAAAAAGCCCAAGACGGACC	TTGAGCTTGGCGCACTTCTT
Sox2	qRTPCR	GATCAGCATGTACCTCCCCG	TCCTCTTTTTGCACCCCTCC
Sox4	qRTPCR	AGAAGGTGAAGTCGGGCAAC	CTGGAGAGAACGATGCAGCC
Sox5	qRTPCR	CTGACGCAAGAGACTTGTGGT	TTCCGCCTTCTGAGGTGAGG
Sox6	qRTPCR	TACAGCTTCAGCTCTATGCCG	TGCTGCTTTTGTTTGGCAGG

Sox8	qRTPCR	TACCCGCATCTCCATAACGC	CGGCCAGTCTTCACACTCTT
Stc2	qRTPCR	AACGTCGGTGTGATTGTGGA	TGACTGCCTCCTTCACATCT
Syt9	qRTPCR	CACGACAGCTGCCAAGATTTC	CAGACCACAGGCAGTAACCA
Tbr1	qRTPCR	CCGAGTCCAGACGTTCACTT	CAGCCCGTGTAGATCGTGT
Tcf4	qRTPCR	GCCATTCGCTGTTGGTGATG	AGAAGCCGCGCGACTATTTC
Tcf7l1	qRTPCR	AGAAGCCGCGCGACTATTTC	AGGAGGGGCCATTTCATCTG
Tcf7l2	qRTPCR	GGCAAGATGGAGGGCTCTTTA	GCACCACCGGTACTTTGTTC
Tfdp2	qRTPCR	ACCGAAAACCTTAGGGCCAA	AGTCTGAGTGACCATTGCGG
Thbs1	qRTPCR	CAACGCCACAGTTCCTGATG	GAACGACCACGTTGCTGAAT
Tm7sf2	qRTPCR	CTCGCCTCGGTTCCTTTGA	GCCATTGACCAGCCACATAG
Tnfrsf10b	qRTPCR	TACACCAGCCATTCCAACCA	CCTGGTTTGCATCGACACAC
Tox4	qRTPCR	CTTGAGGAAGTGACGCGCT	GACAGCCAGTGAGGGATCAG
Vangl2	qRTPCR	GAAACAGGCGAGTGGTCTGG	TTTGCTGGACAAGTGGGCTT
Wdhd1	qRTPCR	GGTGTCGAGCAATAAGGGCA	ACTGGAAGGGCTTTTGTCGT
Wee1	qRTPCR	CCCACGTCGTTCGCTATTTC	CCGGCCAACTTGCAAAAGG
Wnt7a	qRTPCR	GATCAAGCAGAATGCCCGGA	GCCTAGCTCTCGGAACTGTG
Adcy1_Promotor	ChIP-qPCR	CGGGGCCTCTAGTAGGAAGA	CTGAGATCGCTCCCCTGTTC
Crym_Promotor	ChIP-qPCR	AGTGACGCCTAACCTTGACC	GACACCTTACCACTCTGGGG
Cux1_Promotor	ChIP-qPCR	CTGAGCGCTCGTTCCTAAGT	ATCCTGAAACGCGTGCTTTG
Cux2_Promotor	ChIP-qPCR	GCTAGGCAGGGTGTCATGTT	CCGCCCTGGGTTAAAGATCA
Dkk3_Promotor	ChIP-qPCR	CAAGCTGGATCTGGTCACGA	CTGGATACAGGCGCTTTTGC
Pou3f3_Promotor	ChIP-qPCR	AGACAACTGCCGGGGAAAAA	TCACAGTCTGGTGCGACATC
Satb2_Promotor	ChIP-qPCR	TCCTTTTGGGTTGTCCTCGG	CATTGCGGTGGACCAGTCT
Scna_Promotor	ChIP-qPCR	AGCCTTTTCCTTCCCTCAGC	CCAGACGCAACCAAAACAGG
Slit1_Promotor	ChIP-qPCR	GGCCAGAGTGGAGAGAACAC	CCCACATCCCCAACTCTGTC
Sox11_Promotor	ChIP-qPCR	CTCTGTCCATCACCTACGCC	AAATGACCCAGAAGGTGCGT
Sox4_Promotor	ChIP-qPCR	AGCTTAGAGCAGACCCCAAT	AGGAAGCGTTAGTTACAGCGG
Sox5_Promotor	ChIP-qPCR	TACACTTCCAGCAGCGGTCT	GCGCCTGGAGGTTGTCTAA
Syt9_Promotor	ChIP-qPCR	CTTGGCTCTAAACCCCGCAT	TCCAAAGCACGTTCTCGGTT
Tbr1_Promotor	ChIP-qPCR	TCAGCGCGACAGCTTATGAT	AGTTAACCCACCCAGCAACC
Cre	Genotyping	ATGCTTCTGTCCGTTTGCCG	CCTGTTTTGCACGTTCACCG
Dot1l_cKO	Genotyping	GCCTACAGCCTTCATCATTC	CCCATACAGTACTCACCGGAT
NesCre	Genotyping	GCGGTCTGGCAGTAAAAACTATC	GTAGGTGGAAATTCTAGCATCATCC
		CTAGGCCACAGAATTGAAAGATCT	GTGAAACAGCATTGCTGTCACTT
Dot1l_flox	Genotyping	GCCTACAGCCTTCATCATTC	GATAGTCTCAATAATCTCA
Dot1l	ISH-probe	ATTGCTCTTGTTCTCTCGCTGCTGTCTTCGCCTAGCTGCCTCCTGCTCCTCCCT<br>GAGTTTTGGATTTTTCAGACTAGAAAAATAGTTTTCAAGTATGGTGCGGTCAA<br>TGGTGTGCAGATAGTAGGAGACAGGCTTGCCAGTCCACGACACAGAGCCCTT<br>CAGGGGCGACAGCTCCACCACACGCATGATGGTGCCAATGTCACTCAAGTTC<br>CTGCTGTTGATCCTGAAGTTCAGAGGGGCAAAGGGCTTTGAGGATACGATTCT<br>GCCGCCTTCCTTCATGTTTGCGAATCGCTCCTTCAGCTGGTGATCCACCTCAG<br>GACCAAAGGCAAAGTTATTCACAAATATAACACTCGTGTTGGCGATCCGTTC<br>CCTCCATTCCTCTGAGAGGAAGTCACCTCGTTCCAGTGTGTATTCTGCATGCTT<br>TTTTCCATACCATTTCATCCACTTCCTGAACTCTCGGTCCATGGTCTCTGCGTA<br>CTTGGCTGGGATGTCCGCTTTCTCCACTCCGTAGTGATGTTTGCAGTTGGTGG	

Lhx2	ISH-probe	TCCCATATGGTCGACCTGCAGGCGGCCGCActaGTGATTGGCACAGAAGTTAAG<br>ACTGACGTCACAGTCGGCAGGGTGGGCTAGTCAAGTCTGTGAGGGTTGTAG<br>GCGTGCTGGAGGGGCTGAGCGAGGCGTTGGACAGCTCCGAGGCGGGCCCTGA<br>CGGCGTCCCTGTCTGCAGCGTGGCATCTGACGTCTTGTCCACGCCCGTGTTTTC<br>CTGCCGTAAAAGGTTGCGCCTGAACTTGGCCCGGGCATTCTGAAACCAGACCT<br>GGAGGACTCTCTTGGTGAGGCCGGTCTTTTGCGCAAGCTGCTTCAAGTCCTTG<br>GCATCGGGATTGTGGTTAATGGCAAAGTAAGACTTCATTGTCCGAAGCTGGTG<br>GTGCTTGGAGGAGGTGCGCATGCGCTTTGTCTTTTGGCTGCTGGGGTAGGGCT<br>GGTCACGATCCAGGTGTTCAGCATCGTTCTCGTTACAGCTAAGCGCGGCGTTG<br>TAAGCTGCCAGATCTGCCCCGGGTCCTGGACTCTTGCGCTTTCTCGGCCTCCC<br>CTTTTGCACAGTGCCCACGCCGTTGTAGTAGGGAAGACCCAGCGGATTAGCCC<br>CGGCTGAGCCCAATCCTGCACTCTTAGCTGCTGCGGCTGCGGCTGCCGCCGCT<br>GCCACGTCGGCATGGTTAAAGTGTGCTGGGTATTcgCCCTGCAGCAGAGCCTC<br>GAAGTGCAAGCGGCAATAGACCAGGCTGTCCTTCATGCCGAAATGGTCGCCG<br>GTCGTCAGCATCTTGTTACACGTTGTGCATGTGAAGCAGTTGAGGTGATAAAC<br>CAAGTCCCGAGCGCGCATCACCATCTCTGAGGCCGAGATGCCCAGGTGGCAG<br>CGGgCGCAGCGCTGCACAGAGAACCGCCTGTAGTAGTCTTCTTTGCAGTAGAT<br>GCTGCCATCCTTGCTGAAGCAGGTGAGTTCCGACTCCAGGTTGAGCTTGCATT<br>CACAGCACTTGAGGCAGCGCATGTGCCATTGCTTgtcTACTGCCAGCAGGTA
Reln	ISH-probe	CGCGGGatTTCaGCCTAgAgTGcAgCCTTCTGTTTCagGACTAGTCngAGGcTACaG<br>GGCCATCATACTTTGCTGGTGTTGGCTCCACCTCATCAgAAgGtaTGACTATGAC<br>CCACaaGGAAACAACCCACGAAGAGAACACaGTGAGGGAAACATCCaCCATTT<br>TACAAGTCACTTTGTTACCACAgACaCnAAATGGCAGTTACTGTCAGTAAAAC<br>GGcCACagAacacaTTAAAAAATTGTTTTAgACAAGGTAATGGGGACTCCACTGT<br>GgGAGGGAGATAGGGCGTTCaTCCACAATTTTTTAAGTAgAAAATATCTGAAG<br>GTCAAACATTTTTTTTCCTATCcacaaaATGATGCTACATCAACATACGTGTTAA<br>GGAGCATATTAAgGAGACAGCAGTGTCTTTACAGTTATCATCTTGAATAGGTA<br>TCTGCATTAACTTTCTGCATAATACAAGTAGGtCAAAgtCCAgCAgCAAAATAT<br>TGCAaCACAGCAAGTAGATACAGTTTCAGTGTGTTATACGGTAGAGAAGAAT<br>ACgTAAAGGACAAACGACGAGAACTTATTCATATAAACaAGAGACAGAGCTG<br>AAaTACAGTCTACTTAAATAgcTCGGTGACCAACAATGTTAAATAATCACTAgT<br>GCGGCCGCCTGCAgGTCGACCATATGGGAgAGCTCCCAACGCGTTGGATGCAT<br>AGCTTGAGTatTCTATAGTGTCACCTAAATAGCTTGGCGTAATCaTGGTCaTAgc<br>TgnTTCCTGTGtGAAATTGTTATCCGCTCACaATTCCaCACaACATACgAgcCgGA<br>AgCATAAAGtGTAAAgcCtGGGGtGCCTAatGAGtGAgCTAACTCaCATTAATTGCG<br>TtGCGCTCActGnCCGnnTTTCCAGtCGGgAAAnCtGtC

Supplemental Table S2

Antibody	Purpose	Company	Purchase number	Amount
anti-H3	Immunoblot	Abcam, UK	ab12079	1/1000
anti-H3S10ph	Immunofluorescence	Upstate, USA	06 570	1/200
anti-Ki67	Immunofluorescence	Leica, Germany	NCL-ki67p	1/500
anti-TBR2	Immunofluorescence	Abcam, UK	ab23345	1/500
anti-active-CASP3	Immunofluorescence	Cell signaling, USA	9661	1/500
anti-BrdU	BrdU labeling	Abcam, UK	ab1893	1/100
anti-CTIP2	Immunofluorescence	Abcam, UK	ab18465	1/200
anti-GFP	Immunofluorescence	Abcam, UK	ab13970	1/1000
anti-H3K4me3	ChIP	Diagenode, Belgium	pAb-003-050	3µg/ChIP
anti-H3K79me2	Immunoblot, ChIP	Abcam, UK	ab3594	1/1000; 3µg/ChIP
anti-PAX6	Immunofluorescence	DSHB Hybridoma Bank, USA	AX6-s	1/50
anti-RFP	Immunofluorescence	Thermo Fisher Scientific, USA	MA5-15257	1/500
anti-SATB2	Immunofluorescence	Abcam, UK	ab51502	1/100
anti-SOX1	Immunofluorescence	Abcam, UK	ab87775	1/1000
anti-SOX11	Immunofluorescence	University Erlangen-Nürnberg	kind gifts of Dr. Elisabeth Sock	1/500
anti-SOX2	Immunofluorescence	Abcam, UK	ab97959	1/1000
anti-SOX4	Immunofluorescence	University Erlangen-Nürnberg	kind gifts of Dr. Elisabeth Sock	1/1000
anti-SOX5	Immunofluorescence	Abcam, UK	ab94396	1/500
anti-TBR1	Immunofluorescence	Abcam, UK	ab31940	1/200
anti-γ-TUBULIN	Immunofluorescence	GenTex, USA	GTX113286	1/500

Supplemental Table S3

Gene name	log2(fold change) with p-value<0.05	Location	Author	Citation
Lhx5	0,660	L1	Miquelajáuregui A et al	J Neurosci. 2010 Aug 4;30(31):10551-62. doi: 10.1523/JNEUROSCI.5563-09.2010.
Reln	0,958	L1	Sarnat HB et al	Semin Pediatr Neurol. 2002 Dec;9(4):302-8.
Btbd11	-0,621	UL	Bedogni F et al	PNAS July 20, 2010. 107 (29) 13129-13134
Tle1	-0,197	UL	Molyneaux BJ et al	Nat Rev Neurosci. 2007 Jun;8(6):427-37.
Rgs8	-0,649	UL	Lein ES et al	Nature volume 445, pages 168–176 (11 January 2007)
Zfpm2	0,655	UL	Nielsen JV et al	Cereb Cortex. 2014 May;24(5):1216-29. doi: 10.1093/cercor/bhs400. Epub 2013 Jan 2.
Ntng2	0,458	UL	Bedogni F et al	PNAS July 20, 2010. 107 (29) 13129-13134
Satb2	-0,748	UL	Sessa A et al	Neuron. 2008 Oct 9;60(1):56-69. doi: 10.1016/j.neuron.2008.09.028.
Cux1	-0,356	UL	Molyneaux BJ et al	Nat Rev Neurosci. 2007 Jun;8(6):427-37.
Cux2	-1,050	UL	Molyneaux BJ et al	Nat Rev Neurosci. 2007 Jun;8(6):427-37.
Plxnd1	0,677	UL	Molyneaux BJ et al	Nat Rev Neurosci. 2007 Jun;8(6):427-37.
Pou3f3	-0,675	UL	Molyneaux BJ et al	Nat Rev Neurosci. 2007 Jun;8(6):427-37.
Cyp39a1	-0,575	UL	Molyneaux BJ et al	Nat Rev Neurosci. 2007 Jun;8(6):427-37.
Glra2	0,983	UL	Allen Brain Atlas	Allen Brain Atlas
Lrrtm1	0,350	UL	Francks A et al	Mol Psychiatry. 2007 Jul 31. doi: 10.1038/sj.mp.4002053 23.
Gap43	0,406	LL	Jacobs KM et al	J Comp Neurol. 1993 Oct 1;336(1):151-60.
Prnp	0,490	LL	Allen Brain Atlas	Allen Brain Atlas
Snca	1,151	UL-LL	Allen Brain Atlas	Allen Brain Atlas
Sox5	-0,289	LL	Molyneaux BJ et al	Nat Rev Neurosci. 2007 Jun;8(6):427-37.
Syt9	0,473	LL	Molyneaux BJ et al	Nat Rev Neurosci. 2007 Jun;8(6):427-37.
Slit1	-0,633	LL	Whitford KL	Neuron, Volume 33, Issue 1, 3 January 2002, Pages 47-61
Crym	1,687	LL	Molyneaux BJ et al	Nat Rev Neurosci. 2007 Jun;8(6):427-37.
Dkk3	0,847	LL	Molyneaux BJ et al	Nat Rev Neurosci. 2007 Jun;8(6):427-37.
Npy	-0,827	LL	Perrenoud Q et al	Cereb Cortex. 2013 Feb;23(2):423-41. doi: 10.1093/cercor/bhs032. Epub 2012 Feb 22.
Adcy1	0,526	LL	Abdel-Majid RM et al	Nat Genet. 1998 Jul;19(3):289-91.
Mc4r	0,609	LL	Liu H et al	Journal of Neuroscience 6 August 2003, 23 (18) 7143-7154
Fosl2	0,378	LL	Allen Brain Atlas	Allen Brain Atlas
Ppp1r1b	0,629	LL	Bedogni F et al	PNAS July 20, 2010. 107 (29) 13129-13134
Nr4a3	0,585	LL	Molyneaux BJ et al	Nat Rev Neurosci. 2007 Jun;8(6):427-37.
Tbr1	(n.s.) 0,132	LL	Bedogni F et al	PNAS July 20, 2010. 107 (29) 13129-13134
Chst2	0,343	LL	Allen Brain Atlas	Allen Brain Atlas
Sstr2	0,304	SVZ	Allen Brain Atlas	Allen Brain Atlas
Nhlh1	0,462	SVZ/IZ	Ayoub AE et al	Proc Natl Acad Sci U S A. 2011 Sep 6;108(36):14950-5. doi: 10.1073/pnas.1112213108. Epub 2011 Aug 22.
Nfib	-0,430	SVZ	Plachez C et al	J Comp Neurol. 2012 Oct 1;520(14):3135-49. doi: 10.1002/cne.23081.
Sema6d	-0,407	SVZ/IZ	Ayoub AE et al	Proc Natl Acad Sci U S A. 2011 Sep 6;108(36):14950-5. doi: 10.1073/pnas.1112213108. Epub 2011 Aug 22.
Hopx	0,868	SVZ	Thomsen ER et al	Nat Methods. 2016 Jan;13(1):87-93. doi: 10.1038/nmeth.3629. Epub 2015 Nov 16.
Arx	-0,859	VZ	Colasante G et al	Cereb Cortex. 2015 Feb;25(2):322-35. doi: 10.1093/cercor/bht222. Epub 2013 Aug 22.
Hey1	-0,383	VZ/SVZ	Ayoub AE et al	Proc Natl Acad Sci U S A. 2011 Sep 6;108(36):14950-5. doi: 10.1073/pnas.1112213108. Epub 2011 Aug 22.
Prom1	-0,349	VZ/SVZ	Weimer JM et al	Development. 2009 Sep;136(17):2965-75. doi: 10.1242/dev.036616.
Mid1	-0,616	VZ/SVZ	Ayoub AE et al	Proc Natl Acad Sci U S A. 2011 Sep 6;108(36):14950-5. doi: 10.1073/pnas.1112213108. Epub 2011 Aug 22.
Fgfrl1	-0,438	VZ/SVZ	Ayoub AE et al	Proc Natl Acad Sci U S A. 2011 Sep 6;108(36):14950-5. doi: 10.1073/pnas.1112213108. Epub 2011 Aug 22.
Lhx2	-1,176	VZ/SVZ	Molyneaux BJ et al	Nat Rev Neurosci. 2007 Jun;8(6):427-37.
Otx1	-1,103	VZ/SVZ	Molyneaux BJ et al	Nat Rev Neurosci. 2007 Jun;8(6):427-37.
Cst3	0,471	VZ	Ayoub AE et al	Proc Natl Acad Sci U S A. 2011 Sep 6;108(36):14950-5. doi: 10.1073/pnas.1112213108. Epub 2011 Aug 22.
Stxbp6	0,536	VZ	Ayoub AE et al	Proc Natl Acad Sci U S A. 2011 Sep 6;108(36):14950-5. doi: 10.1073/pnas.1112213108. Epub 2011 Aug 22.
Slc1a3	0,390	VZ/SVZ	Ayoub AE et al	Proc Natl Acad Sci U S A. 2011 Sep 6;108(36):14950-5. doi: 10.1073/pnas.1112213108. Epub 2011 Aug 22.
Ednrb	0,404	VZ/SVZ	Ayoub AE et al	Proc Natl Acad Sci U S A. 2011 Sep 6;108(36):14950-5. doi: 10.1073/pnas.1112213108. Epub 2011 Aug 22.
Wdhd1	-0,293	VZ/SVZ	Ayoub AE et al	Proc Natl Acad Sci U S A. 2011 Sep 6;108(36):14950-5. doi: 10.1073/pnas.1112213108. Epub 2011 Aug 22.
Igf2bp1	-1,269	VZ/SVZ	Ayoub AE et al	Proc Natl Acad Sci U S A. 2011 Sep 6;108(36):14950-5. doi: 10.1073/pnas.1112213108. Epub 2011 Aug 22.

L1	layer 1
UL	upper layer neurons
LL	lower layer neurons
IZ	intermediate zone
SVZ	subventricular zone
VZ	ventricular zone
n.s.	not significant

FIGURE LEGENDS TO SUPPLEMENTAL MATERIAL

Figure S1: ***Dot1l*-cKO^{Emx1} leads to impaired cortical and hippocampal development.** A) Nissl stained sections of P0 ctrl and *Emx1*-Cre *Dot1l*-cKO brains. Black squares in upper panels represent higher magnification images depicted below. Annotations: Cortical plate (CP), intermediate zone (IZ), subventricular/ventricular zone (SVZ/VZ), cornu ammonis (CA), dentate gyrus (DG). Scale bars: 200µm.

Figure S2: **Loss of DOT1L increases *Reln* and reduces *Lhx2* expression domain. A)** ISH to visualize expression of *Reelin* (*Reln*) in E14.5 ctrl and *Foxg1-Cre Dot1l*-cKO forebrains; upper panel gives an overview; lower panel shows magnification of the cortical plate. Scale bars as indicated in the figures. **B)** ISH to visualize expression of *Lhx2* in E14.5 ctrl and *Foxg1-Cre Dot1l*-cKO forebrains; upper panel gives an overview; lower panel shows magnification of the cortical plate. Scale bars as indicated in the figures. **C)** Example of quantification approach for immunostainings of markers for different cortical layers in tissue samples with different radial dimensions, exemplified for PAX6 immunostaining at E14.5 of ctrl animals. Representation of the grid encompassing the subdivision into 10 bins and 200µm width of the cortex (left). The grid was fitted to each individual tissue section to overlay the complete developing cortex, keeping equal bin sizes within one section but allowing for variable bin sizes in different sections of the same animal or between animals. Using this procedure, variations in the cortical size were considered in the quantifications. The number of cells was determined using Cell counter plug-in of ImageJ (right), and normalized to the area. Results of cell countings were represented as the number of cells/mm^2 in each bin. **D)** Same as in C, but shown for PAX6 staining at E18.5.

Figure S3: Loss of DOT1L does not activate ER stress transcriptional programs or apoptosis *in vivo*. A) RNA-seq expression data (*Foxg1-Cre Dot1l*-cKO vs ctrl) of ER stress genes (left) and qRTPCR validation (right) of the genes implicated in ER stress in *Dot1l*-cKO animals normalized to ctrl (n=3). **B)** and **D)** Representative immunostainings of activated CASP3 (aCASP3) of E14.5 (B) and E18.5 (D) ctrl and *Dot1l*-cKO forebrains. Insets in B show positive staining in trigeminal ganglion as positive control for the staining. Scale bar: 200µm. **C)** and **E)** Quantification of the total number of aCASP3-positive cells in the cortex and hippocampus at E14.5 (C) and E18.5 (E) (n=4). Data are presented as mean ± SEM. P-values were calculated using unpaired, two tailed Student's t-test: *p<0.05, **p<0.01, ***p<0.001.

Figure S4: *Emx1-Cre Dot1l* conditional knockout leads to a decreased numbers of progenitors and UL neurons alongside altered distribution of DL neurons. A) Left: Representative immunostainings of E18.5 forebrains for neural progenitor marker PAX6 and TBR2, DL markers TBR1 and CITP2, and UL marker SATB2 (all red) and DAPI (blue) for ctrl and *Emx1-Cre Dot1l*-cKO. Scale bar: 50µm. Right: Quantification of number of stained cells/mm^2 within each individual bin out of 10 bins spanning the entire cortex from VZ (bin 10) to the marginal zone (bin 1) in a width of 200µm (n=4 for all markers and genotypes). **B)** Total numbers of PAX6, TBR2, TBR1, CTIP2, or SATB2-positive cells at E18.5 in 200µm width of the cortex, as determined from the sum of positive cells for the respective markers counted in A comparing ctrl (white bars) and *Emx1-Cre Dot1l*-cKO (grey bars). All data are presented as mean ± SEM. P-values were calculated using unpaired, two tailed Student's t-test: *p<0.05, **p<0.01, ***p<0.001.

Figure S5: H3K79me2 profiles at DE and non-DE genes and transcriptional alterations in *Foxg1-Cre* heterozygote forebrains. A) H3K79me2 coverage (log_2(ChIP/Input)) of all DE and non-DE genes at E14.5 as revealed by ChIP-seq of wild type telencephalon, encompassing the gene body, from TSS to TES, and a range of +/-4Kb flanking the gene body. This shows enrichment of H3K79me2 at DE genes compared to non-DE genes. **B)** Venn-diagram to represent the overlap between DE genes of ctrl vs *Dot1l*-cKO at E14.5, with DE genes of heterozygote *Foxg1-Cre* vs ctrl forebrains at E13.5. **C)** Heatmaps representing H3K79me2 enrichment (blue: strong enrichment, red: few enrichment) and distribution

for the 132 overlapping genes indicated in B, encompassing the gene body, from TSS to TES, and a range of +/-4Kb flanking the gene body and clustered in four k-means clusters. Data derived from ChIP-seq analysis of wild type E14.5 forebrains.

Figure S6: Loss of DOT1L influences the cell cycle. A) WikiPathway analysis of genes with decreased expression upon *Dot1l*-cKO. WikiPathway groups were arranged from top to bottom according to the given p-value (lowest p-value on top). **B)** Left: RNA-seq data (white bars, *Foxg1-Cre Dot1l*-cKO vs ctrl), and qRTPCR validations (grey bars, normalized to ctrl, n=3) for genes involved in cell cycle regulation (indicated by classification on the left) and with altered expression levels upon *Dot1l*-cKO. Right: High resolution representation of H3K79me2 enrichment (in $\log_2$(ChIP/Input)) and distribution in wild type forebrains at E14.5. **C)** Quantification of the number of cells positive for M-phase marker H3S10ph (n=4) in *Foxg1-Cre Dot1l*-cKO vs ctrl E14.5 and E18.5 cortical sections; normalized to the number of positive cells quantified in control animals, which was set as 100% (dashed line). **D)** Representative immunoblot showing the levels of H3K79me2 after 48hs pharmacological inhibition of DOT1L using two different inhibitors, SGC0946 and EPZ5676, respectively, or DMSO (ctrl) of neural progenitor cells (NPC) derived *in vitro* from mESCs. Total histone H3 served as loading control. **E)** Densitometric analysis of immunoblots of H3K79me2 after 48hs pharmacological inhibition of DOT1L in NPC normalized to DMSO treated ctrl (n=4). Data are presented as mean ± SEM. P-values were calculated using unpaired, two tailed Student's t-test: *p<0.05, **p<0.01, ***p<0.001.

Figure S7: Expression of UL genes *Satb2*, *Pou3f3*, *Cux1* and *Cux2* during mouse brain development. A) qRTPCR gene expression analysis of indicated genes at different stages of development and in adult stage, in cortical tissue, normalized to E11.5 (E11.5-E17.5 n=3, adult n=4). Data are presented as mean ± SEM. P-values were calculated using unpaired, two tailed Student's t-test: *p<0.05, **p<0.01, ***p<0.001.

Figure S8: *In utero* electroporation of DOT1L OE plasmid at E14.5 and control of tamoxifen induced recombination in *Nes-Cre Dot1l*-cKO animals. A) Upper panel: Representative image of immunostainings of cortical sections from E14.5 animals for PAX6, TBR2, TBR1, CTIP2, or SATB2 (green), RFP (red) and DAPI (blue) within their main expression domains, after *in utero* electroporation of DOT1L overexpression plasmid at E12.5. RFP marks electroporated cells, which assumingly overexpress DOT1L. Lower panel: Quantification of electroporated cells (RFP) that co-expressed PAX6, TBR2, TBR1, CTIP2, and SATB2, given as percentage of the total number of RFP/DOT1L OE cortical cells within the main expression domains/layers of the respective layer marker at E14.5 (SATB2 n=3, CTIP2 n=3, TBR1 n=3, TBR2 n=3 and PAX6 n=3(ctrl)/4(*Dot1l*-cKO)). Data are presented as mean ± SEM. **B)** Representative immunostainings of ctrl and *Nes-Cre Dot1l*-cKO E18.5 forebrains for GFP without (no Tmx) or after three single tamoxifen (Tmx) injections at consecutive days starting at E9.5, E11.5, or E13.5, respectively, to control for recombination activity of CRE. Scale bar: 50µm. **C)** Summary of phenotypic alterations of cortical development after interference with DOT1L expression in three different mouse models and at different time points during development, as indicated through differences in marker expression of PAX6, TBR2, TBR1, CTIP2, and SATB2 of *Foxg1-Cre*, *Emx1-Cre* and *Nes-Cre Dot1l*-cKO mice at three different time points. Light green ovals: number of cells expressing the respective marker decreased significantly in the respective mouse model. Dark green ovals: reduced numbers of cells expressing the respective marker, which did not reach significance. Orange squares: significantly altered distribution of cells within the cortical plate. Orange squares with green striped ovals: increased numbers of cells expressing the respective marker and altered distribution in the cortical plate. n.a.: not analyzed; n.c.: no change.

Table S1: List of used primers and probes
Table S2: List of used antibodies
Table S3: References for gene expression in the developing cortex and in specific layers

SUPPLEMENTAL METHODS

In situ hybridization, Hematoxylin-Eosin staining and immunofluorescence

ISH, HE staining and immunostainings of brain tissue and cultured cells was performed as previously described (1, 2). For ISH, probes listed in supplemental table S1 were applied. Antibodies applied are listed in supplemental table 2.

RNA extraction, cDNA synthesis, quantitative real-time PCR and analysis

RNA was extracted from E14.5 control (ctrl) and *Foxg1-Cre Dot1l*-cKO dorsal telencephalon for RNA-seq. For qRTPCR, we dissected the dorsal and ventral telencephalon and used the dorsal samples if not stated otherwise. For E12.5 we isolated the entire cerebral cortex for RNA extraction. For analyses of expression changes in NPC differentiated from mESC, we harvested 3 million cells in PBS.

For RNA extraction, samples were processed with QIAshredder Kit (#79654, Qiagen, Germany) and RNAeasy Mini Kit (#74104, Qiagen, Germany) including an on-column DNAse digestion (#79254, Qiagen, Germany). cDNA synthesis, qRTPCR (primers listed in supplemental table S1) and analysis were performed as described (1). Data are presented as mean ± SEM. Pairwise analyses were conducted by unpaired, two-tailed Student's t tests using GraphPad Prism 6. Significance is indicated in the figure legends.

Imaging and quantifications

Immunofluorescence images were obtained using an Axioplan M2 fluorescent microscope (Zeiss) equipped with an Apotome.2 module. Using Adobe Illustrator, a grid was placed over the image of the cortex. The grid was 200µm wide and contained 10 equally sized bins in the radial dimension (3). The total number of cells or total number of cells/bin were counted using ImageJ and expressed as number of cells in 200µm (example in Figs. S2C, D). For statistical analysis, 1 to 2 cortices of one animal were counted and pooled for at least 3 animals. In each figure legend the N number for each marker quantified specified. In the case that a different n was used for controls and *Dot1l*-cKO this is stated as n = n(ctrl)/n(cKO). The cell number was normalized to the height of the cortex resulting in cells/mm^2. Means from different biological replicates were compared using unpaired Student's t-test using GraphPad Prism 6. Each bin from the ctrl condition was compared to the corresponding bin of the *Dot1l*-cKO. Y-TUBULIN stained sections were scanned and imaged with a SP8 laser scanning confocal microscope (Leica). Angles of cleavage were measured using the angle tool in ImageJ as previously described by (4). To assess RFP and specific layer marker-positive cells we used the software QuPath (5).

In vivo BrdU labeling and staining

Brain fixation, embedding, BrdU labeling and staining was performed as described (supplemental table S2, (2)).

Protein extraction and immunoblot

3 million NPCs, differentiated from mESC, were lysed using RIPA-buffer and subjected to immunoblotting as described (6). Antibodies used are listed in supplemental table S2. Images were quantified as published (6). Statistical analysis was performed using one column t-test with GraphPad Prism 6. Data are presented as mean ± SEM. Significance is indicated in the figure legends.

mESC cell culture and neuronal differentiation

mESC were differentiated into neuronal lineage according to published protocols (7).

Bioinformatics of RNA-seq and ChIP-seq

Raw data from the Illumina HiSeq 2500 sequencing machine was demultiplexed and converted into FASTQ files using Illumina bcl2fastq2 (version 1.8.4, http://support.illumina.com/downloads/bcl2fastq_conversion_software_184.html),

screened for contamination with fastq_screen (version 0.5.1, (http://www.bioinformatics.babraham.ac.uk/projects/fastq_screen/), and checked for overall read quality (FastQC v0.11.3, http://www.bioinformatics.babraham.ac.uk/projects/fastqc/).

For RNA-seq an automatic detection and trimming of adapters were done using TrimGalore (version 0.2.8, http://www.bioinformatics.babraham.ac.uk/projects/trim_galore/), which is based on cutadapt (8) (minimum overlap with adaptor: 5 bases). 3' end bases with Phred quality score less than 28 were excluded. We used TopHat2 (version 2.0.9(9)) to align the reads against mouse genome build mm10. We did a transcriptome guided mapping with annotations from Ensembl FTP release 79 (10) . For TopHat2, we specified --library-type fr-firststrand, --mate-inner-dist 0 and --mate-std-dev 80 options. Then using htseq-count (version 0.6.0 (8)) we did a strand-specific read counting with option --stranded reverse. Differential gene expression analysis was performed using the DESeq2 (version 1.6.1 (11)) with default settings. We considered the genes with adjusted p-value less than 0.05 as significantly differentially expressed. The whole bioinformatics analysis was carried out on the Galaxy platform (12).

For analysis of the paired-end ChIP-seq libraries, reads were directly mapped with Bowtie2 version 2.2.0 (13) without trimming. The reference genome assembly was GRCm38/mm10; the reference annotation was Ensembl FTP release 79. Picard MarkDuplicates (http://broadinstitute.github.io/picard) was used to remove read duplicates before peak calling with MACS2 version 2.1.0 (14), where the 'broad' option was set for H3K79me2 only. The differential binding analysis was done with DiffBind (15). For all other in-depth ChIP-seq specific analysis deepTools2 version 2.3.5 or 2.4.1 was used (16) i.e. to generate coverage track files (bamCoverage, bamCompare, normalization 1x), estimate the ChIP performance (plotFingerprint), or compare marks between samples (computeMatrix, plotHeatmap). Results from differential expression RNA-seq analysis were also integrated into heatmaps by converting fold-change values into a plotHeatmap input file (custom script) to allow plotting them alongside to the ChIP samples. For the presented heatmaps (Fig. 2A) the 2 groups-K-mean clustering on H3K4me3 (E14.5) and H3K79me2 (E14.5) levels was carried out for *Dot1l*-cKO increased and decreased gene expressions independently and then combined for an overall overview resulting in 4 different clusters. For each TSS (+/- 250bp) of each differentially expressed gene the log2-ratio value was calculated by comparing ChIP to input tracks using bamCompare (deepTools 2.5.3). The values are visualized in the scatterplot and boxplot grouping them by ChIP-seq clusters combined with color-coding (up or down regulation) from RNA-seq results (log2-fold changes).

Figures

The preparation of all final figures were done by using FIJI (ImageJ, v. 2.0.0-rc-43/1.51d)(17), Inkscape (v. 0.91) and Adobe Illustrator.

1. Vezzali,R., Weise,S.C., Hellbach,N., Machado,V., Heidrich,S. and Vogel,T. (2016) The FOXG1/FOXO/SMAD network balances proliferation and differentiation of cortical progenitors and activates Kcnh3 expression in mature neurons. *Oncotarget*, **7**, 37436–37455.

2. Hellbach,N., Weise,S.C., Vezzali,R., Wahane,S.D., Heidrich,S., Roidl,D., Pruszak,J., Esser,J.S. and Vogel,T. (2014) Neural deletion of Tgfbr2 impairs angiogenesis through an altered secretome. *Hum. Mol. Genet.*, **23**, 6177–6190.

3. Pla,R., Borrell,V., Flames,N. and Marín,O. (2006) Layer Acquisition by Cortical GABAergic Interneurons Is Independent of Reelin Signaling. *J. Neurosci.*, **26**, 6924–6934.

4. Pilaz,L.-J., McMahon,J.J., Miller,E.E., Lennox,A.L., Suzuki,A., Salmon,E. and Silver,D.L. (2016) Prolonged Mitosis of Neural Progenitors Alters Cell Fate in the Developing Brain. *Neuron*, **89**, 83–99.

5. Bankhead,P., Loughrey,M.B., Fernández,J.A., Dombrowski,Y., McArt,D.G., Dunne,P.D., McQuaid,S., Gray,R.T., Murray,L.J., Coleman,H.G., *et al.* (2017) QuPath: Open source software for digital pathology image analysis. *Sci. Rep.*, **7**, 16878.

6. Roidl,D., Hellbach,N., Bovio,P.P., Villarreal,A., Heidrich,S., Nestel,S., Grüning,B.A., Boenisch,U. and Vogel,T. (2016) DOT1L Activity Promotes Proliferation and Protects Cortical Neural Stem Cells from Activation of ATF4-DDIT3-Mediated ER Stress In Vitro. *Stem Cells Dayt. Ohio*, **34**, 233–245.

7. Bibel,M., Richter,J., Lacroix,E. and Barde,Y.-A. (2007) Generation of a defined and uniform population of CNS progenitors and neurons from mouse embryonic stem cells. *Nat. Protoc.*, **2**, 1034–1043.

8. Martin,M. (2011) Cutadapt removes adapter sequences from high-throughput sequencing reads. *EMBnet.journal*, **17**, 10–12.

9. Kim,D., Pertea,G., Trapnell,C., Pimentel,H., Kelley,R. and Salzberg,S.L. (2013) TopHat2: accurate alignment of transcriptomes in the presence of insertions, deletions and gene fusions. *Genome Biol.*, **14**, R36.

10. Amonida (2015) Ensembl 79 has been released! *Ensembl Blog*.

11. Anders,S., Pyl,P.T. and Huber,W. (2015) HTSeq—a Python framework to work with high-throughput sequencing data. *Bioinformatics*, **31**, 166–169.

12. Afgan,E., Baker,D., van den Beek,M., Blankenberg,D., Bouvier,D., Čech,M., Chilton,J., Clements,D., Coraor,N., Eberhard,C., *et al.* (2016) The Galaxy platform for accessible, reproducible and collaborative biomedical analyses: 2016 update. *Nucleic Acids Res.*, **44**, W3–W10.

13. Langmead,B. and Salzberg,S.L. (2012) Fast gapped-read alignment with Bowtie 2. *Nat. Methods*, **9**, 357–359.

14. Zhang,Y., Liu,T., Meyer,C.A., Eeckhoute,J., Johnson,D.S., Bernstein,B.E., Nusbaum,C., Myers,R.M., Brown,M., Li,W., *et al.* (2008) Model-based Analysis of ChIP-Seq (MACS). *Genome Biol.*, **9**, R137.

15. Ross-Innes,C.S., Stark,R., Teschendorff,A.E., Holmes,K.A., Ali,H.R., Dunning,M.J., Brown,G.D., Gojis,O., Ellis,I.O., Green,A.R., *et al.* (2012) Differential oestrogen receptor binding is associated with clinical outcome in breast cancer. *Nature*, **481**, 389–393.

16. Ramírez,F., Ryan,D.P., Grüning,B., Bhardwaj,V., Kilpert,F., Richter,A.S., Heyne,S., Dündar,F. and Manke,T. (2016) deepTools2: a next generation web server for deep-sequencing data analysis. *Nucleic Acids Res.*, **44**, W160–W165.

17. Schindelin,J., Arganda-Carreras,I., Frise,E., Kaynig,V., Longair,M., Pietzsch,T., Preibisch,S., Rueden,C., Saalfeld,S., Schmid,B., *et al.* (2012) Fiji: an open-source platform for biological-image analysis. *Nat. Methods*, **9**, 676–682.
